# CONSORT 2010 statement: extension to randomised pilot and feasibility trials

**DOI:** 10.1186/s40814-016-0105-8

**Published:** 2016-10-21

**Authors:** Sandra M. Eldridge, Claire L. Chan, Michael J. Campbell, Christine M. Bond, Sally Hopewell, Lehana Thabane, Gillian A. Lancaster, on behalf of the PAFS consensus group, Doug Altman, Frank Bretz, Marion Campbell, Erik Cobo, Peter Craig, Peter Davidson, Trish Groves, Freedom Gumedze, Jenny Hewison, Allison Hirst, Pat Hoddinott, Sarah E. Lamb, Tom Lang, Elaine McColl, Alicia O’Cathain, Daniel R. Shanahan, Chris Sutton, Peter Tugwell

**Affiliations:** 1 Centre for Primary Care and Public Health, Queen Mary University of London, London, UK; 2 School of Health and Related Research, University of Sheffield, Sheffield, UK; 3 Centre of Academic Primary Care, University of Aberdeen, Aberdeen, Scotland, UK; 4 Nuffield Department of Orthopaedics, Rheumatology and Musculoskeletal Sciences, University of Oxford, Oxford, UK; 5 Clinical Epidemiology and Biostatistics, McMaster University, Hamilton, Ontario Canada; 6 Department of Mathematics and Statistics, Lancaster University, Lancaster, UK

## Abstract

The Consolidated Standards of Reporting Trials (CONSORT) statement is a guideline designed to improve the transparency and quality of the reporting of randomised controlled trials (RCTs). In this article we present an extension to that statement for randomised pilot and feasibility trials conducted in advance of a future definitive RCT. The checklist applies to any randomised study in which a future definitive RCT, or part of it, is conducted on a smaller scale, regardless of its design (eg, cluster, factorial, crossover) or the terms used by authors to describe the study (eg, pilot, feasibility, trial, study). The extension does not directly apply to internal pilot studies built into the design of a main trial, non-randomised pilot and feasibility studies, or phase II studies, but these studies all have some similarities to randomised pilot and feasibility studies and so many of the principles might also apply.

The development of the extension was motivated by the growing number of studies described as feasibility or pilot studies and by research that has identified weaknesses in their reporting and conduct. We followed recommended good practice to develop the extension, including carrying out a Delphi survey, holding a consensus meeting and research team meetings, and piloting the checklist.

The aims and objectives of pilot and feasibility randomised studies differ from those of other randomised trials. Consequently, although much of the information to be reported in these trials is similar to those in randomised controlled trials (RCTs) assessing effectiveness and efficacy, there are some key differences in the type of information and in the appropriate interpretation of standard CONSORT reporting items. We have retained some of the original CONSORT statement items, but most have been adapted, some removed, and new items added. The new items cover how participants were identified and consent obtained; if applicable, the prespecified criteria used to judge whether or how to proceed with a future definitive RCT; if relevant, other important unintended consequences; implications for progression from pilot to future definitive RCT, including any proposed amendments; and ethical approval or approval by a research review committee confirmed with a reference number.

This article includes the 26 item checklist, a separate checklist for the abstract, a template for a CONSORT flowchart for these studies, and an explanation of the changes made and supporting examples. We believe that routine use of this proposed extension to the CONSORT statement will result in improvements in the reporting of pilot trials.

Editor’s note: In order to encourage its wide dissemination this article is freely accessible on *the BMJ* and *Pilot and Feasibility Studies* journal websites.

**Electronic supplementary material:**

The online version of this article (doi:10.1186/s40814-016-0105-8) contains supplementary material, which is available to authorized users.

## Introduction

The Consolidated Standards of Reporting Trials (CONSORT) statement (www.consort-statement.org) is a guideline designed to improve the transparency and quality of the reporting of randomised trials. It was first published in 1996, revised in 2001, last updated in 2010 [1, 2] and published simultaneously in 10 leading medical journals, including the *Lancet*, *JAMA*, *BMJ*, *Annals of Internal Medicine*, and *PLoS Medicine*. The CONSORT statement comprises a checklist of the minimum essential items that should be included in reports of randomised trials and a diagram documenting the flow of participants through the trial.

The development of CONSORT guidelines has received considerable international recognition. The CONSORT statement has been cited more than 8000 times and has received support from the World Association of Medical Editors, Council of Science Editors, International Committee of Medical Journal Editors, and more than 600 journals worldwide. Several studies have examined the impact of the statement on the reporting quality of published randomised trials and found that adoption of the statement leads to an increase in reporting quality [3].

In addition to the CONSORT statement, extensions to the CONSORT checklist for reporting trials with non-inferiority, equivalence, and cluster or pragmatic designs have been published [4–6], as have extension checklists for reporting harms [7], different types of interventions (non-drug treatments [8] and herbal interventions [9]), and patient reported outcomes [10]. The main CONSORT statement and all of the current extensions focus on trials for which the research question centres on the effectiveness or efficacy of an intervention. However, some randomised trials, that we refer to as pilot and feasibility trials, do not have effectiveness or efficacy as their primary focus. Rather, they are designed to support the development of a future definitive RCT. By “definitive” in this context we mean an appropriately powered study focusing on effectiveness or efficacy. The need for high standards in conduct and reporting applies just as much to pilot and feasibility trials as it does to definitive trials.

## Scope of this paper

In this article we present an extension to the CONSORT statement for randomised pilot and feasibility trials conducted in advance of a future definitive RCT. In keeping with the broad scope of CONSORT, the future definitive RCT might evaluate either the efficacy or the effectiveness of an intervention. The primary aim of the randomised pilot or feasibility trial, however, is to assess feasibility of conducting the future definitive RCT.

We make no distinction in this extension between pilot and feasibility randomised trials. Although in practice we recognise that different researchers might have preferences for different terms, the lack of distinction is based on a framework developed by the authors, which defines such studies [11]. In that framework, a feasibility study for a future definitive RCT asks whether the future trial can be done, should be done, and, if so, how. Pilot studies are a subset of feasibility studies. They ask the same questions about feasibility (whether the future trial can be done, should be done, and, if so, how) but have a particular design feature: in a pilot study (that might or might not be randomised) the future definitive RCT, or part of it, is conducted on a smaller scale.

For brevity, we use the term “pilot trial” to refer to any randomised study in which a future definitive RCT, or a part of it, is conducted on a smaller scale. However, these studies might legitimately be referred to using any of the following terms: pilot RCT, randomised pilot trial, pilot trial, pilot study, randomised pilot study, feasibility RCT, randomised feasibility trial, feasibility trial, feasibility study, or randomised feasibility study. In fact, we have set no restrictions on the terminology used to describe pilot trials; rather we have specified only that they are randomised, conducted in advance of a future definitive RCT, and primarily aim to assess feasibility.

The development of this extension was motivated by the growing number of studies described as feasibility or pilot studies [12] and by research that has identified weaknesses in the reporting and conduct of these studies [12–15]. We expect that improved reporting quality will lead to more high quality examples of pilot trials, enabling yet further improvements in the conduct of pilot trials and making it possible for readers to use the results of reported pilot studies in preparing future trials in similar settings and with similar participants. Because the purpose of a pilot trial (to assess feasibility) is different from that of the future definitive RCT (to assess effectiveness or efficacy), the focus of the reporting should be different, and that difference is reflected in the extension.

The extension does not apply to internal pilot studies that are built into the design of a main trial, or to non-randomised pilot and feasibility studies. However, much of what is presented here might apply to, or be adapted to apply to, these types of pilot or feasibility studies or similar types of trial, such as “proof of concept” or phase II trials done in the development of drugs [16, 17]. Proof of concept or phase II trials are small RCTs the main objective of which are to inform the sponsor whether or not to continue the development of a drug with larger trials. Similar to pilot trials, the focus is on assessing the feasibility of further development rather than assessing effectiveness or efficacy. However, to do this these trials tend to focus on aspects such as safety and potential effectiveness or efficacy. They might use accepted methods devised for phase II trials [18] to assess the outcome to be used in a future phase III trial (which could be meta-analysed if required) [19] or use surrogate outcomes—that is, intermediate measures, often biochemical, which have less direct impact on a patient than, for example, cure or death, but which should be associated with these “hard” outcomes. Safety, and potential effectiveness or efficacy, are usually less important in pilot trials, where the focus is on the development of interventions and their evaluation and where issues related to feasibility might be different. Nevertheless, pilot trials do sometimes assess potential effectiveness using surrogate outcomes. For example, oxygenation of the blood as a surrogate measure for improved lung function and survival [20] or the number of steps walked each day as a surrogate for clinical measures of heart disease [21].

Here we present an extension to the standard CONSORT guidelines for reporting RCTs. Many investigators, however, use qualitative research alongside other methods to assess feasibility. The amount of qualitative work conducted at the pilot and feasibility stage, its relation with any pilot trial, and the way investigators want to report this work, varies. Stand-alone qualitative studies that are reported separately from the pilot trial, such as Hoddinott et al. and Schoultz et al. [22, 23] should follow appropriate reporting guidelines [24–26] and should provide link references to other pilot work carried out in preparation for the same definitive trial. When qualitative work is reported within the primary report of a pilot trial [27], it is not always possible to put sufficient detail into the methods section of the report to comply with reporting guidelines for qualitative studies. If this is the case, we recommend an online supplement or appendix to report the methods in detail. O’Cathain et al., Hoddinott et al., and Schoultz et al. have provided guidelines and examples for conducting qualitative feasibility studies alongside pilot trials [22, 23, 26, 28, 29].

## Adapting the CONSORT statement for pilot trials

The development of this CONSORT extension for pilot trials is described briefly here and in detail elsewhere [30]. Before developing the checklist for this extension, the research team agreed on the definitions of pilot and feasibility studies. This was done by initially considering pilot and feasibility studies to be discrete types of study and therefore in need of separate checklists. However, preliminary work concluded that pilot and feasibility studies could not be defined in a mutually exclusive way, compatible with current understanding and the use of these terms among the research community. We therefore adopted an overarching definition of feasibility studies, with pilot studies being a subset, and developed a single checklist for such studies that use a randomised approach, referred to as pilot trials in this paper. The process of agreeing on the definitions of feasibility and pilot studies and the underpinning conceptual framework are reported separately [11]. That work was done in parallel with the development of the checklist (Table 1). We used the principles in Box 1 to guide the work.

**Table 1:** Stages of adapting CONSORT statement for pilot trials

Stage	Activity	Participants	Venue (or virtual meeting)	Date
1	Drafting of definitions and preliminary adaptation of CONSORT checklist items	Research team	London	Dec 2012
2	1st round of modified Delphi process using online administration	Invited experts from research community (trialists, methodologists, statisticians, funders, and journal editors)	Email distribution	Jul-Aug 2013
3	2nd round of modified Delphi process	As for round 1	Email distribution	Sept-Oct 2013
4	Review of results from Delphi process and redrafting checklist	Research team	London	Feb 2014
5	Consensus meeting	Invited experts (trialists, methodologists, statisticians, funders, journal editors, and members of CONSORT executive)	Oxford	Oct 2014
6	Review of consensus meeting feedback and drafting final checklist	Research team	Email consultation with consensus participants; and meetings in London	Dec 2014-Dec 2015; and Jan, Jun, Dec 2015
7	Further review and piloting	Research team	Email consultation with consensus participants; and piloting by independent researchers writing up pilot studies	Mar 2016; and Jan-Mar 2016

In stage 1, the research team met and worked through each of the existing CONSORT checklist items, agreeing whether each was relevant and should be retained, not relevant and should be excluded, or needed rewording in the context of either a feasibility study or pilot study. This resulted in two checklists. We then applied the revised checklists to a sample of 30 articles identified from previous work [13, 15] and our own personal collections.

In stage 2, we used a modified Delphi survey to seek consensus on the appropriateness of each of the checklist items. Participants (*n* = 93) were asked to rate each item on a scale of 1 to 9 (1 = not at all appropriate to 9 = completely appropriate). They were also given the opportunity to comment on each item, definitions of pilot and feasibility studies, and the perceived usefulness of the checklist [11].

In stage 3, participants in the Delphi survey were asked to review responses for items that 70% or more of participants had rated as 8 or 9 in round 1 of the survey and to make additional comments on these items. They were asked to review the remaining items and classify each using one of four options: discard, keep, unsure, or no opinion. They were also asked to add any items they believed had been missed. In total 93/120 (77.5%) responses were received for round 1 and 79/93 (84.9%) for round 2.

In stage 4, the research team met face to face to review the feedback from the Delphi survey and to revise the checklist. In stage 5, the revised checklist was then further reviewed in detail during a two day expert consensus meeting. In stage 6, some checklist items were reworded to ensure clarity of meaning and purpose, and the research team met face to face a further three times to agree on the final wording of the checklist, identify examples of good reporting, and develop the explanation and elaboration section of this paper. A full draft of the paper was then sent to members of the consensus meeting to ensure it fully reflected the discussion of the meeting.

Table 2 presents the final checklist, laid out in accordance with other CONSORT extensions. Items in the standard checklist column should be adhered to unless the extension column indicates a change in the item. Box 1 lists the methodological considerations and principles that guided the process.

**Table 2:** CONSORT checklist of information to include when reporting a pilot trial

Section/topic and item No	Standard checklist item	Extension for pilot trials	Page No where item is reported
Title and abstract			
1a	Identification as a randomised trial in the title	Identification as a pilot or feasibility randomised trial in the title	
1b	Structured summary of trial design, methods, results, and conclusions (for specific guidance see CONSORT for abstracts)	Structured summary of pilot trial design, methods, results, and conclusions (for specific guidance see CONSORT abstract extension for pilot trials)	
Introduction			
Background and objectives:			
2a	Scientific background and explanation of rationale	Scientific background and explanation of rationale for future definitive trial, and reasons for randomised pilot trial	
2b	Specific objectives or hypotheses	Specific objectives or research questions for pilot trial	
Methods			
Trial design:			
3a	Description of trial design (such as parallel, factorial) including allocation ratio	Description of pilot trial design (such as parallel, factorial) including allocation ratio	
3b	Important changes to methods after trial commencement (such as eligibility criteria), with reasons	Important changes to methods after pilot trial commencement (such as eligibility criteria), with reasons	
Participants:			
4a	Eligibility criteria for participants		
4b	Settings and locations where the data were collected		
4c		How participants were identified and consented	
Interventions:			
5	The interventions for each group with sufficient details to allow replication, including how and when they were actually administered		
Outcomes:			
6a	Completely defined prespecified primary and secondary outcome measures, including how and when they were assessed	Completely defined prespecified assessments or measurements to address each pilot trial objective specified in 2b, including how and when they were assessed	
6b	Any changes to trial outcomes after the trial commenced, with reasons	Any changes to pilot trial assessments or measurements after the pilot trial commenced, with reasons	
6c		If applicable, prespecified criteria used to judge whether, or how, to proceed with future definitive trial	
Sample size:			
7a	How sample size was determined	Rationale for numbers in the pilot trial	
7b	When applicable, explanation of any interim analyses and stopping guidelines		
Randomisation:			
Sequence generation:			
8a	Method used to generate the random allocation sequence		
8b	Type of randomisation; details of any restriction (such as blocking and block size)	Type of randomisation(s); details of any restriction (such as blocking and block size)	
Allocation concealment mechanism:			
9	Mechanism used to implement the random allocation sequence (such as sequentially numbered containers), describing any steps taken to conceal the sequence until interventions were assigned		
Implementation:			
10	Who generated the random allocation sequence, enrolled participants, and assigned participants to interventions		
Blinding:			
11a	If done, who was blinded after assignment to interventions (eg, participants, care providers, those assessing outcomes) and how		
11b	If relevant, description of the similarity of interventions		
Analytical methods:			
12a	Statistical methods used to compare groups for primary and secondary outcomes	Methods used to address each pilot trial objective whether qualitative or quantitative	
12b	Methods for additional analyses, such as subgroup analyses and adjusted analyses	Not applicable	
Results			
Participant flow (a diagram is strongly recommended):			
13a	For each group, the numbers of participants who were randomly assigned, received intended treatment, and were analysed for the primary outcome	For each group, the numbers of participants who were approached and/or assessed for eligibility, randomly assigned, received intended treatment, and were assessed for each objective	
13b	For each group, losses and exclusions after randomisation, together with reasons		
Recruitment:			
14a	Dates defining the periods of recruitment and follow-up		
14b	Why the trial ended or was stopped	Why the pilot trial ended or was stopped	
Baseline data:			
15	A table showing baseline demographic and clinical characteristics for each group		
Numbers analysed:			
16	For each group, number of participants (denominator) included in each analysis and whether the analysis was by original assigned groups	For each objective, number of participants (denominator) included in each analysis. If relevant, these numbers should be by randomised group	
Outcomes and estimation:			
17a	For each primary and secondary outcome, results for each group, and the estimated effect size and its precision (such as 95% confidence interval)	For each objective, results including expressions of uncertainty (such as 95% confidence interval) for any estimates. If relevant, these results should be by randomised group	
17b	For binary outcomes, presentation of both absolute and relative effect sizes is recommended	Not applicable	
Ancillary analyses:			
18	Results of any other analyses performed, including subgroup analyses and adjusted analyses, distinguishing prespecified from exploratory	Results of any other analyses performed that could be used to inform the future definitive trial	
Harms:			
19	All important harms or unintended effects in each group (for specific guidance see CONSORT for harms)		
19a		If relevant, other important unintended consequences	
Discussion			
Limitations:			
20	Trial limitations, addressing sources of potential bias, imprecision, and, if relevant, multiplicity of analyses	Pilot trial limitations, addressing sources of potential bias and remaining uncertainty about feasibility	
Generalisability:			
21	Generalisability (external validity, applicability) of the trial findings	Generalisability (applicability) of pilot trial methods and findings to future definitive trial and other studies	
Interpretation:			
22	Interpretation consistent with results, balancing benefits and harms, and considering other relevant evidence	Interpretation consistent with pilot trial objectives and findings, balancing potential benefits and harms, and considering other relevant evidence	
22a		Implications for progression from pilot to future definitive trial, including any proposed amendments	
Other information			
Registration:			
23	Registration number and name of trial registry	Registration number for pilot trial and name of trial registry	
Protocol:			
24	Where the full trial protocol can be accessed, if available	Where the pilot trial protocol can be accessed, if available	
Funding:			
25	Sources of funding and other support (such as supply of drugs), role of funders		
26		Ethical approval or approval by research review committee, confirmed with reference number	

## Extension of CONSORT 2010 to pilot trials

### Title and abstract

- Item 1a

- *Standard CONSORT item*: identification as a randomised trial in the title

- *Extension for pilot trials*: identification as a pilot or feasibility randomised trial in the title

- *Example 1 (using the words pilot, randomised, and trial)*

− “Bespoke smoking cessation for people with severe mental ill health (SCIMITAR): a pilot randomised controlled trial” [31]

- *Example 2 (using the words feasibility, randomised, and trial)*

− “A cluster randomised feasibility trial evaluating nutritional interventions in the treatment of malnutrition in care home adult residents” [32]

- *Explanation*

The primary focus of these guidelines is randomised pilot and feasibility trials. To ensure that these types of studies can be easily identified from specific search criteria, a title containing the descriptors “pilot” or “feasibility” as well as “randomised” provides a necessary, recognised terminology for selecting randomised pilot and feasibility trials [13]. This would also enable these studies to be easily indexed in electronic databases, such as PubMed [33]. Although the descriptors might appear in the title for many studies, they might not necessarily occur together, as in: “Feasibility of a randomised trial of a continuing medical education program in shared decision-making on the use of antibiotics for acute respiratory infections in primary care: the DECISION+ pilot trial [34].” Furthermore, in some cases authors might use the phrase “randomised pilot study” or “randomised feasibility study,” as in “‘Not just another walking program’: Everyday Activity Supports You (EASY) model—a randomized pilot study for a parallel randomized controlled trial [21].” Such papers could be identified in appropriate searches. However, in general we recommend the descriptors are given together in one phrase, and the word “trial” rather than “study” is used, as in “randomised pilot trial” or “randomised feasibility trial.”

- Item 1b

- *Standard CONSORT item*: structured summary of trial design, methods, results, and conclusions (for specific guidance see CONSORT for abstracts) [35, 36].

- *Extension for pilot trials*: structured summary of pilot trial design, methods, results, and conclusions (for specific guidance see CONSORT abstract extension for pilot trials) (Table 3)

**Table 3:** Extension of CONSORT for abstracts for reporting pilot trials

Item	Standard checklist item	Extension for pilot trials
Title	Identification of study as randomised	Identification of study as randomised pilot or feasibility trial
Trial design	Description of the trial design (eg, parallel, cluster, non-inferiority)	Description of pilot trial design (eg, parallel, cluster)
Methods:		
Participants	Eligibility criteria for participants and the settings where the data were collected	Eligibility criteria for participants and the settings where the pilot trial was conducted
Interventions	Interventions intended for each group	
Objective	Specific objective or hypothesis	Specific objectives of the pilot trial
Outcome	Clearly defined primary outcome for this report	Prespecified assessment or measurement to address the pilot trial objectives*
Randomisation	How participants were allocated to interventions	
Blinding (masking)	Whether or not participants, caregivers, and those assessing the outcomes were blinded to group assignment	
Results:		
Numbers randomised	Number of participants randomised to each group	Number of participants screened and randomised to each group for the pilot trial objectives*
Recruitment	Trial status†	
Numbers analysed	Number of participants analysed in each group	Number of participants analysed in each group for the pilot objectives*
Outcome	For the primary outcome, a result for each group and the estimated effect size and its precision	Results for the pilot objectives, including any expressions of uncertainty*
Harms	Important adverse events or side effects	
Conclusions	General interpretation of the results	General interpretation of the results of pilot trial and their implications for the future definitive trial
Trial registration	Registration number and name of trial register	Registration number for pilot trial and name of trial register
Funding	Source of funding	Source of funding for pilot trial

*Space permitting, list all pilot trial objectives and give the results for each. Otherwise, report those that are a priori agreed as the most important to the decision to proceed with the future definitive RCT

†For conference abstracts

- *Example*

**Fig 1:**
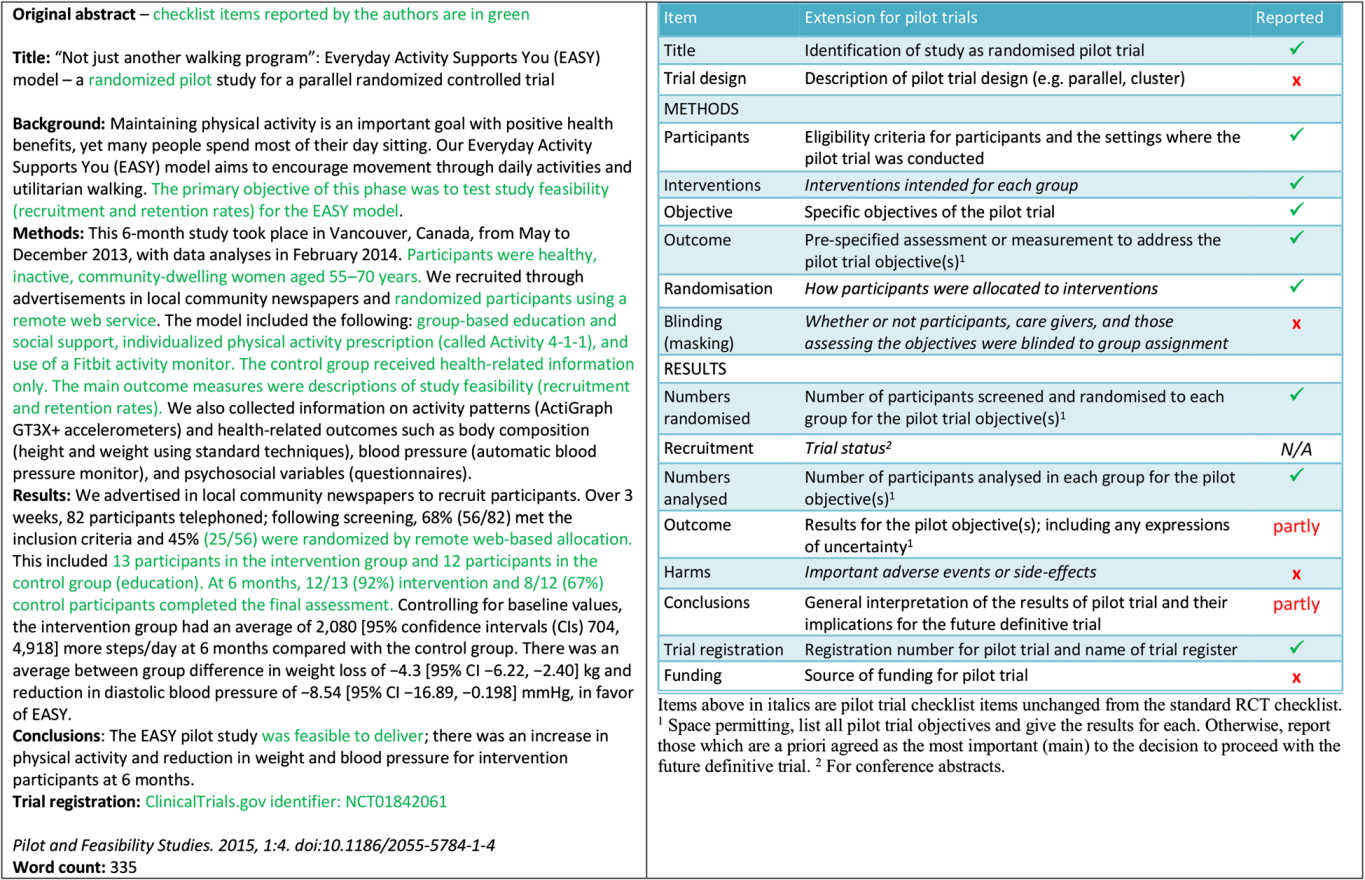
Example of abstract for report of pilot trial [21], shown alongside CONSORT for abstracts extension for pilot randomised trials

**Fig 2:**
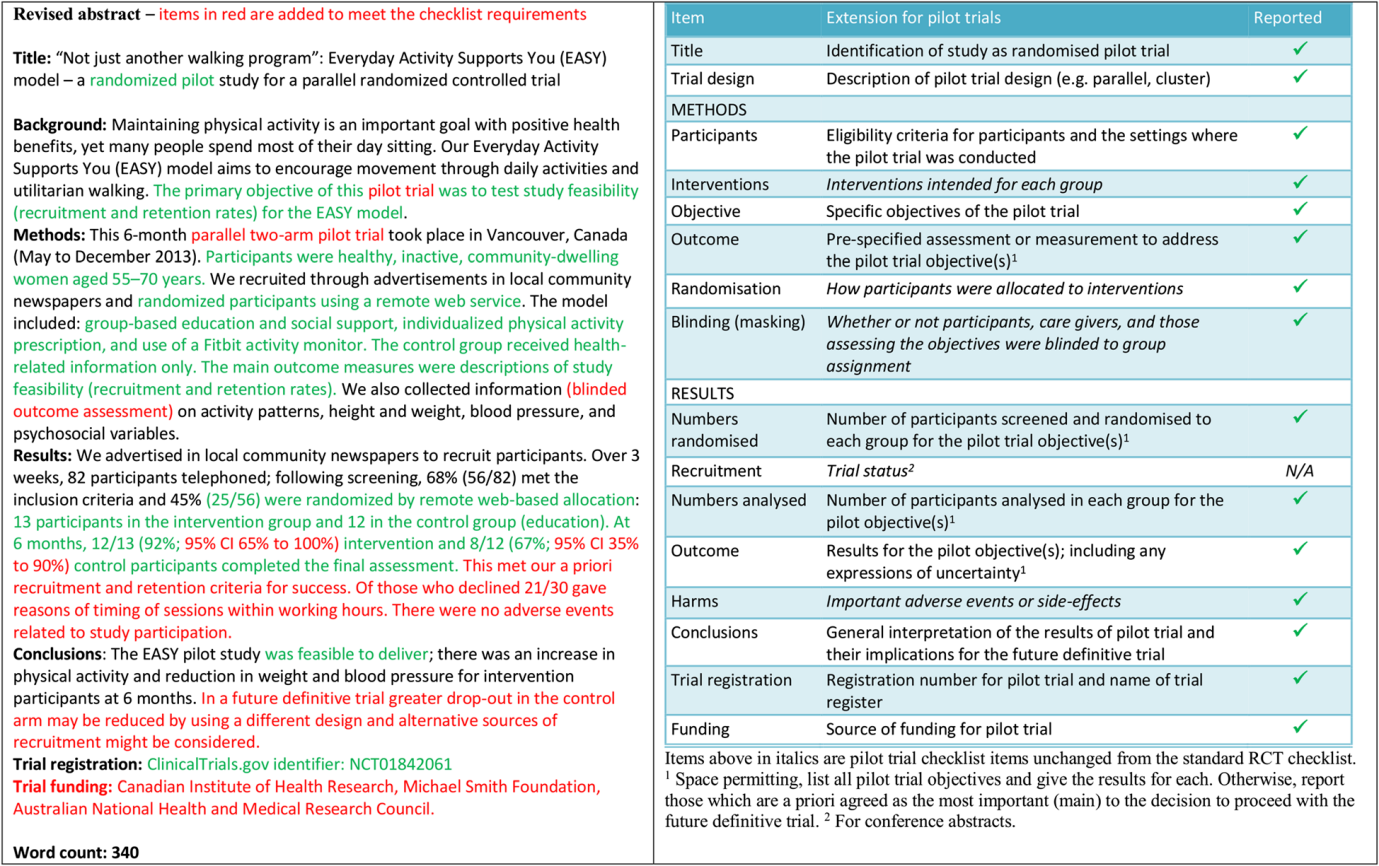
Revised version of example abstract for report of pilot trial [21], shown alongside CONSORT for abstracts extension for pilot randomised trials

**Fig 3:**
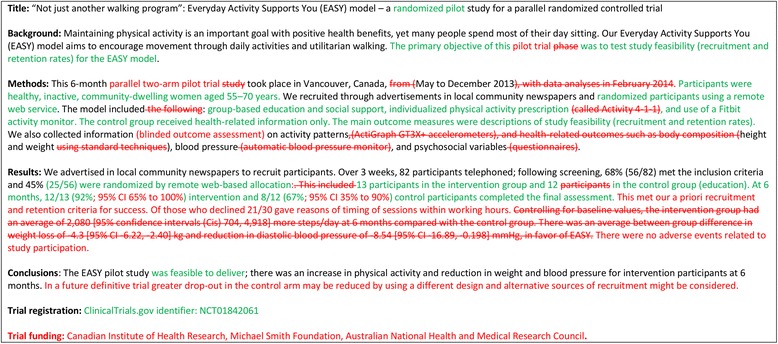
Track changes version of example abstract for report of pilot trial [21], showing changes between Figs. 1 and 2

See Figs. 1, 2 and 3 [21].

- *Explanation*

Abstracts can follow different structures dependent on a journal’s style. They are typically around 300 words. We outline what information should be reported in the abstract irrespective of style. This information may also be used for writing conference abstracts. The structure of the abstract does not differ in format from item 1b of the standard CONSORT 2010 guidelines. However, its content focuses on the aims and objectives of the pilot trial and not on the future definitive RCT.

It is important that the abstract contains pertinent information on the background, methods, results, and conclusions in relation to the feasibility objectives and outcomes, and that it states the study is a “randomised” pilot trial. This will aid researchers in understanding the nature of the paper and facilitates electronic searching through the inclusion of specific key words. A statement in the abstract that this study is in preparation for a future definitive RCT is recommended to place it in context. A description of the areas of uncertainty to be addressed and a statement of the feasibility aims and objectives should be included in the background, how these objectives have been addressed in the methods, and results for each objective in the results. If there are a limited number of pilot trial objectives then all should be listed and results for each reported. If there are many pilot trial objectives, then agreement should be reached a priori about which are the most important, to decide whether to proceed to a future definitive RCT, and only these objectives should be reported. An explicit statement relating to whether the future definitive RCT is likely to go ahead on the basis of the results of the pilot trial should also form part of the discussion and conclusions.

### Introduction

- Item 2a

- *Standard CONSORT item*: scientific background and explanation of rationale

- *Extension for pilot trials*: scientific background and explanation of rationale for future definitive trial, and reasons for randomised pilot trial

- *Example*

“Reduced fetal movements (RFM) is a frequently seen problem in maternity care with 6–15% of women reporting attending at least one occasion of RFM to health professionals in the third trimester of pregnancy. RFM, defined by maternal perception of significantly reduced or absent fetal activity, is associated with increased risk of stillbirth and fetal growth restriction (FGR) due to placental dysfunction. Despite this association there is a paucity of evidence to direct clinical management of women presenting with RFM. This has been recently highlighted by guidelines from the Royal College of Obstetrics and Gynaecology (RCOG) and a meta-analysis…The absence of high-quality evidence has led to wide variation in management strategies for RFM in high-income settings…Although there are randomised controlled trials (RCT) of counting fetal movements by a formal structure (e.g. count to ten) there have been no published RCTs of patient management following presentation with RFM. To undertake an RCT of patient management raises important practical concerns including: maternal anxiety for fetal wellbeing, the need to make a decision regarding participation in a short period of time due to the acute nature of RFM and adherence to protocol. Thus, studies have adopted an approach of changing practice at the unit level in quality-improvement projects or stepwise cluster RCT (AFFIRM, NCT01777022). We performed this study to address whether an RCT of the management of RFM in individual patients was an appropriate trial design, and was feasible with regard to i) maternal recruitment and retention ii) patient acceptability, iii) adherence to protocol. In addition, we wished to confirm the prevalence of poor perinatal outcomes in the study population [37].”

- *Explanation*

It is important that the scientific background sets the scene and gives the rationale and justification for the future definitive RCT and why the pilot trial is needed, because under the principles of the Helsinki declaration it is unethical to expose people unnecessarily to the risks of research [38]. The background and rationale are nicely illustrated in the example. Other related publications, or preliminary work such as systematic reviews, qualitative studies, or additional feasibility work, or absence of such work because no one has looked at this topic before, should also be mentioned. The rationale for the randomised pilot trial should be clearly outlined, including the areas of uncertainty that need to be addressed before the future definitive RCT can take place and why such a trial is needed before proceeding to the future definitive RCT. This rationale is usually reported in the final paragraph of the introduction or background section to provide a justification for the pilot trial.

- Item 2b

- *Standard CONSORT item*: specific objectives or hypotheses

- *Extension for pilot trials*: specific objectives or research questions for pilot trial

- *Example 1 (listing objectives as primary and secondary)*

“In this feasibility trial, the research aim was to explore trial design, staff and resident acceptability of the interventions and outcome measures and to provide data to estimate the parameters required to design a definitive RCT…The primary objectives of the trial were as follows:

1. To assess how many care homes accepted the invitation to participate in research.
2. To determine whether the eligibility criteria for care home residents were too open or too restrictive by estimating feasible eligibility and recruitment rate.
3. To assess retention of care homes and residents by estimating 3 and 6-month follow-up rates.
4. To investigate the acceptability of nutritional support interventions to malnourished care home residents in terms of compliance and to care home staff in terms of adherence to the intervention schedule.
5. To assess the acceptability and feasibility (and factors influencing this) of the outcome measures as methods to measure efficacy of the interventions within a definitive trial.

The secondary objectives of the trial were as follows:

1. To investigate the completion of screening tools and questionnaires by care home staff.
2. To determine how many malnourished residents were able to participate in PROMs and to complete the questionnaires.
3. To pilot a Healthcare resource usage (HCRU) questionnaire.
4. To measure key outcome domains (for completion rates, missing data, estimates, variances and 95% confidence intervals for the difference between the intervention arms) for malnourished care home residents, including physical outcome measures and PROMs.
5. To collect and synthesise data, from which the Intracluster Correlation Coefficient (ICC) and sample size of a definitive cluster RCT (CRCT) could be estimated [32].”

- *Example 2 (objectives leading to a mixed methods study)*

“The main aim of the study is to assess the feasibility of conducting a definitive trial in terms of recruitment, use and acceptability of the intervention, follow-up at 3 and 6 months, and data collection methods. In addition, the study aims to establish suitable procedures for delivering the intervention and conducting assessments and procedures for ensuring recruitment and retention in the study. Finally, the study aims to discover whether using a structured, individualized approach to lifestyle assessment and referral will improve uptake and participation in lifestyle- and behaviour-change interventions.

The study will also examine, qualitatively, the acceptability of the assessment tool to patients in an acute cardiology setting as well as patients’ experiences of making lifestyle changes in order to develop effective recruitment and retention strategies.

The study will have a number of quantitative objectives:

1. To determine how many patients accept referral to a formal lifestyle programme;
2. To determine how many patients participate in a lifestyle-change intervention or initiate self-managed change;
3. To investigate the uptake of lifestyle intervention in relation to subsequent behaviour change and impact on health-related quality of life, mood and social satisfaction;
4. To estimate feasible eligibility, recruitment and refusal rates, and 3- and 6-months follow-up rates;
5. To measure key outcome domains (that is, for completion rates, missing data, estimates, variances and 95% confidence intervals for the difference between the control and intervention groups) for patients including clinical indicators and patient-reported measures of social satisfaction; health-related quality of life; and mood;
6. To synthesize data to inform the sample size of a definitive trial;
7. To determine the acceptability (and factors influencing this) of financial incentives as a method to encourage behaviour change, their pricing and factors influencing this [39].”

- *Explanation*

Although many aspects of feasibility may be related to each other, an articulation of specific objectives enables readers to understand the main areas of uncertainty to be addressed in the pilot trial and provides a working structure for presenting the methods and results in relation to these objectives. In addition, a comprehensive list of objectives enables other researchers to learn from and adopt similar approaches in their own studies.

It might be beneficial to separate the objectives into primary objectives (often those on which decisions about progressing to a future definitive RCT may be made) and secondary objectives, as in example 1, where feasibility objectives are primary and questions related to patient centred outcomes are treated as secondary. Because it is not always necessary to collect data on patient centred outcomes, it is important to give the rationale for collecting such data. For example, the purpose may be to ensure that certain data can be collected, including from specific patient groups (eg, elderly people, as in example 1), or to ensure that difficult-to-measure concepts such as lifestyle behaviour change can be assessed appropriately in the future definitive RCT (example 2). It might also be informative to state explicitly which objectives will be answered using quantitative methods and which using qualitative methods, as in example 2.

In example 2 the list of quantitative objectives are quite informative, but they are taken from the published study protocol. In the published pilot trial the objectives contained far less detail: “The Healthy Hospital Trial is a single-center, randomized controlled, 2-arm, parallel-group, unblinded feasibility trial that was conducted on 2 cardiology wards at the Leeds Teaching Hospitals Trust. Its primary aim was to explore the feasibility of individualized lifestyle referral assessment, estimate the rate of recruitment, and explore the feasibility of collecting the data and follow-up of participants to inform the sample size of a definitive trial. A secondary aim was to test the concept that an individually tailored assessment improves uptake of lifestyle change compared with usual assessment. The trial protocol has been published elsewhere [40].” We recommend putting detailed individual objectives into the pilot trial report itself so that readers can more easily judge the extent to which these have been fulfilled by the study.

Inclusion of an objective to test a hypothesis of effectiveness (or efficacy) is not recommended (see Box 1). However, other kinds of hypotheses may be tested, such as when using an interim or surrogate outcome to address potential effectiveness [41]. (See also the section entitled Scope of this paper). However, a trial should always be adequately powered for any hypothesis test, and in a pilot trial it should be clearly stated that the objective is to assess potential effectiveness. If tests are carried out without adequate power (as they sometimes are in reality), they should certainly be viewed as secondary and a caveat included in the discussion [21].

### Methods

- Item 3a

- *Standard CONSORT item*: description of trial design (such as parallel, factorial) including allocation ratio

- *Extension for pilot trials*: description of pilot trial design (such as parallel, factorial) including allocation ratio

- *Example*

“We conducted a parallel-group randomised controlled pilot trial… An unequal randomisation of 2:1 vs 1:1 was chosen to provide experience delivering the hydration intervention to more patients [42].”

- *Explanation*

The design of any study should be described, be it a definitive trial or a pilot trial. It is not uncommon for pilot trials to adopt ratios other than the usual 1:1 for randomisation. 1:1 randomisation provides the greatest power for testing effectiveness in, for example, a future definitive RCT. However, a pilot trial commonly involves new, not established, interventions and one of the aims might then be to gain experience in delivering the intervention, in which case it is often better to have as many participants receiving the intervention as is feasible.

- Item 3b

- *Standard CONSORT item*: important changes to methods after trial commencement (such as eligibility criteria), with reasons

- *Extension for pilot trials*: important changes to methods after pilot trial commencement (such as eligibility criteria), with reasons

- *Example*

“After randomly assigning 11 patients (5 to standard care), we recognized that patients assigned to standard care were receiving early surgery because, having achieved accelerated medical clearance, they were put on the operating room list. We therefore amended the protocol to randomly assign patients immediately on diagnosis; only those assigned to early surgery received an expedited medical assessment [43].”

- *Explanation*

Pilot trials are exploratory and so those conducting them should be able to modify the methods if a potential problem becomes apparent. In the case of Buse et al. [43], the original protocol specified that patients had to have medical clearance for rapid surgery before randomisation, but this led to contamination of the control group as some patients in this group were put on the surgical list for rapid surgery (accelerated surgery was the intervention) because it had been ascertained that they were suitable candidates. In the revised protocol participants were randomised first and then assessed for suitability to accelerated access. Thus the pilot potentially improved the design of the trial that was to follow. It is important to document all changes and give reasons for the changes. The example describes changes to the timing of randomisation, but there might also be changes to other aspects of the trial, such as the treatment regimen, eligibility criteria, or outcome variables.

- Item 4a

- *Standard CONSORT item*: eligibility criteria for participants

- *Example*

“Thirty-one sequential eligible people with HD [Huntington’s disease] were recruited from the specialist HD clinics in Cardiff, the United Kingdom, and Oxford, the United Kingdom, between March 2011 and November 2011. Inclusion criteria were (1) diagnosis of HD, confirmed by genetic testing and neurological examination, (2) ability to walk independently as primary means of mobility, (3) willing to travel to the exercise center for the intervention, (4) capacity to give informed consent, (5) Unified Huntington’s Disease Rating Scale Total Motor Score (UHDRS-TMS) and Total Functional Capacity (TFC) of at least 5/124 and 5/13, respectively, from last clinic visit, and (6) maintenance of a stable medical regimen for 4 weeks prior to initiation of study and considered by the recruiting clinician as able to maintain a stable regimen for the course of the study. Participants were not eligible if they (1) had a history of additional prior major neurological condition such as stroke, (2) had an orthopedic condition that limited mobility, (3) demonstrated uncontrolled psychiatric symptoms, (4) were pregnant, (5) demonstrated any contraindication to exercise, or (6) were involved in any interventional trial or within 3 months of completing an interventional trial [44].”

- *Explanation*

Readers might want to know how the results of the trial are likely to apply to the future definitive RCT and other future trials with similar participants in similar settings. A variety of participants (eg, patients, doctors, assessors, caregivers, managers) might provide data to address objectives. For example, in a study in nursing homes, residents were interviewed to seek views on acceptability of the intervention, whereas nurses participated in focus groups to elicit views on randomisation or adherence to treatment protocol [32]. Eligibility criteria should be specified for each set of participants included in a pilot trial. The details provided must be specific enough to identify the clinical population and any other populations and the setting from which they were recruited and to confirm that legal issues were complied with, such as having capacity to give informed consent. Details should be sufficient to allow other researchers to interpret, learn from, and use the information provided.

- Item 4b

- *Standard CONSORT item*: settings and locations where the data were collected

- *Example*

“High-risk adolescents were recruited from three sources: (1) a sample of 205 offspring of BP parents between 12 and 18 years of age enrolled in the NIMH-funded Bipolar Offspring Study at the University of Pittsburgh (BIOS, PI: Birmaher); (2) offspring of adults receiving treatment for BP at Western Psychiatric Institute and Clinic (WPIC); and (3) siblings of youth receiving treatment for BP at the Child and Adolescent Bipolar Services clinic (CABS) at WPIC [45].”

- *Explanation*

The settings for recruiting patients and collecting data must be specified so that readers can judge the applicability (generalisability) of the findings to other trials as well as to the future definitive RCT. Authors should also make clear whether any pilot sites have particular features—for example, organisational features, characteristics that predispose the site to early adoption of new schemes, or specific relationships with the authors that could affect recruitment, consent, and follow-up. This is because these features may not be replicable in other sites and hence in future trials. As with item 4a, details must be sufficient to allow other researchers to interpret, learn from, and use the information.

- Item 4c

- *Extension for pilot trials*: how participants were identified and consented

- *Example*

“Between May and October 2013, clinical staff at participating gastroenterology outpatient clinics scanned and identified potential participants that met the study inclusion criteria. Then, either study invitation packs were sent to patients with researchers’ contact details or patients seen consecutively in clinics were approached with the study information. All study information was co-designed with patients from the patient-involvement group. Interested participants then registered their interest with the researcher by telephone or email. This was followed up with a screening visit with the researcher and then informed written consent was obtained [46].”

- *Explanation*

This is a new item. It is especially important to report details of identification and consent in a pilot trial to allow the feasibility of the recruitment methods to be assessed. The way participants are identified and approached should be described in detail (eg, by advertisement, or selection from medical records or another dataset) to enable readers to understand the generalisability (applicability) of the results. This might be of particular importance for scaling-up for the future definitive RCT, as well as being informative for other future trials. In addition, it is important to know of any specific aspects that might not be easy to implement in the future definitive RCT. Furthermore, a view is sometimes held that pilot trials do not need to be as rigorous in their processes as other trials, so it might be particularly important in these trials to show rigorous and ethical identification and recruitment processes. If details of the way participants were identified and consent obtained are already published in a protocol, then this should be clearly referenced.

- Item 5

- *Standard CONSORT item*: the interventions for each group with sufficient details to allow replication, including how and when they were actually administered

- *Example*

“Intervention (EXERcise or STRETCHing)

The amount of time required for participating in the exercise activities was the same for the EXER group and the STRETCH group. The only difference was the amount of energy expended during the activity. At the first session, the exercise trainer explained the procedures for the respective intervention (EXER or STRETCH), showed them the equipment available for the exercise or stretch sessions, and the coordinator familiarized the participant with the Actical device. The first two weeks required a minimum of 3 sessions at CI [Cooper Institute] for the trainer to teach them how to use the equipment and complete the exercise or stretch routines. Following the first 2 weeks, participants began doing their exercise program at home or other location (gym, park, etc.), and only had to come to CI once a week for an exercise session. Each EXER/STRETCH session averaged about 30–40 min.

EXERcise Intervention

Supervised exercise sessions at the Cooper Institute (CI) for the participants began by using the treadmills or stationary cycles. The CI trainers also taught patients how to complete home-based exercise sessions (e.g., choice of Wii Sports and Fit, jazzercise, jogging, weight training based on their preferred exercise) that were unsupervised workouts at the patient's home or in the community. The duration of each session generally was the time required to reach 1/3 or 1/4 of the total weekly caloric expenditure. There was a progression to the assigned exercise dose in the first few weeks that got them up to their minimum of 12 kilocalories/kilogram/week (KKW) energy expenditure (e.g., 8 KKW first week, 10 KKW second and 12 KKW by the third week). Participants exercised three times per week.

STRETCH Intervention

The stretch group spent approximately the same amount of time, but at energy expenditures of less than 4 KKW per session. After two weeks of three sessions at CI they moved to once a week at CI and two home-based sessions. A 5–10 minute stretching warm-up period included stretches that exercise the major muscle groups of the body. The series included such traditional “warm-up” stretches as: stretches of the gluts, inner thigh, calves and ankles, Achilles tendon, hamstring stretches, shoulder rolls forward and back, shoulder shrugs, isometrics for the neck hugging knees into the chest, moving forehead to right knee, then to left, then to both, and use of the pelvic tilt. An additional 10–15 minutes consisted of moving on to right and left calf stretches, quad stretches, and then to a series for the arms, hands, fingers, wrist, biceps/triceps, shoulders and back. All of the exercises were designed to be done slowly, emphasizing proper alignment, and rest periods to minimize overall physical exertion while obtaining general flexibility, and most importantly controlling for contact time with trainers and any social facilitation from participating in such activities. We had a different set of low level/low intensity routines for each of the 12 weeks to minimize boredom with the routines [47].”

- *Explanation*

If the pilot trial is to inform future research, the authors should report exact details of the treatment given to all study groups, and if one group receives treatment as usual this should also be described thoroughly. Details should include who administered the treatment, as well as what it comprised and how often and where it was delivered. The template for intervention description and replication (TIDieR) guidelines should be followed and the checklist completed [48]. If there are changes to the details of the treatments for any group, these must be reported (see item 3b).

- Item 6a

- *Standard CONSORT item*: completely defined prespecified primary and secondary outcome measures, including how and when they were assessed

- *Extension for pilot trials*: completely defined prespecified assessments or measurements to address each pilot trial objective specified in item 2b, including how and when they were assessed

- *Example*

*“Acceptability* and *demand* were assessed in terms of the usage and repeated usage of the intervention by the patients in the trial indicated by logged user statistics. The interventions’ *practicability* was considered as the ability to log in and occurrence of constraints in delivery and was assessed in terms of the percentage of users in adolescents and professionals, its bounce percentage (percentage of login-errors) and other login-problems. The bounce-percentage was logged and participants were asked to report login-errors. *Integration* was assessed in terms of the extent to which our web-based intervention promotes care that was consistent with recognized standards of diabetes care for adolescents including those published by the International Diabetes Federation (IDF) in collaboration with the International Society for Pediatric and Adolescent Diabetes (ISPAD) and the American Diabetes Association (ADA; 3, 33); see also Appendix 1 [49].”

- *Explanation*

In a definitive trial investigators are primarily interested in response variables or outcomes that enable them to fulfil the primary objective (to assess the effect of an intervention or treatment), and a clear articulation of prespecified outcomes is required to guard against bias in the assessment of this effect. In a pilot trial, however, objectives should relate to feasibility (see Box 1 and item 2b) and any measurements or assessments should enable these objectives to be addressed. To ensure the pilot trial meets its objectives, measures or assessments should be defined to address each separate objective or research question. In the example, objectives were to assess acceptability, demand, practicability, and integration. The authors list the measures used for each of these.

Variables that might be considered primary and secondary outcomes for the future definitive RCT might be measured in a pilot trial to assess response, completeness, or validity. The appropriate measures or assessments would then be response rates, completion rates, or measures of validity. Sometimes investigators may want to measure surrogate outcomes (see example in item 7b), variables on the causal pathway of what might eventually be the primary outcome in the future definitive RCT, or outcomes at early time points, in order to assess the potential for the intervention to affect likely outcomes in the future definitive RCT (see item 2b).

- Item 6b

- *Standard CONSORT item*: any changes to trial outcomes after the trial commenced, with reasons

- *Extension for pilot trials*: any changes to pilot trial assessments or measurements after the pilot trial commenced, with reasons

- *Example 1 (change to assessment time period)*

“Our outcome measures examined uptake and cessation because we hoped that our intervention would affect uptake by referring more people and the success rate of those referred by supporting adherence to treatment…The intervention had two distinct phases so, although not planned in the protocol, we examined uptake of services and 4-week quit rates by trial arm, in these two periods [50].”

- *Example 2 (change to measurement instrument)*

“We defined…initiation of change as participation in a formal program or a self-directed program that was intended to result in change either in diet, physical activity, smoking, or alcohol consumption at any time (binary)…In our published protocol, we had proposed 4 categories of change, but we found it difficult to distinguish between “persisted” and “maintained” in the qualitative follow-up interviews; hence, we combined persistence and maintenance of change in 1 category [40].”

- *Explanation*

An assessment or measure might change during a pilot trial because the change enables investigators to glean more information about the operation of the intervention (as in example 1) or for reasons of acceptability or practicability (example 2). In example 2 it became impractical to use a measurement instrument with four categories when it was identified that researchers could not distinguish between two of the categories. In the interests of full reporting and because of the usefulness of such information to others working in the same specialty, all such changes should be reported.

- Item 6c

- *Extension for pilot trials*: if applicable, prespecified criteria used to judge whether, or how, to proceed with future definitive trial

- *Example*

“Feasibility (delivery) and acceptability (uptake) of the DECISION+ program were the main outcome measures of this pilot trial. Investigators had established a priori threshold for specific feasibility and acceptability criteria. These were the following: (a) the proportion of contacted FMGs [Family medicine groups] participating in the pilot study would be 50% or greater, (b) the proportion of recruited family physicians participating in all three workshops would be 70% or greater, (c) the mean level of satisfaction from family physicians regarding the workshops would be 65% or greater, and (d) the proportion of missing data in each completed questionnaire would be less than 10% [34].”

- *Explanation*

This is a new item. The purpose of a pilot trial is to assess the feasibility of proceeding to the next stage in the research process. To do this investigators need some criteria on which to base the decision about whether or not to proceed. The next stage in the research process will normally, although not always, be the future definitive RCT.

The UK National Institute for Health Research requires that pilot or feasibility studies have clear criteria for deciding whether or not to progress to the next stage: “We expect that when pilot or feasibility studies are proposed by applicants, or specified in commissioning briefs, a clear route of progression criteria to the substantive study will be described. Listing clear progression criteria will apply whether the brief or proposal describes just the preliminary study or both together. Whether preliminary and main studies are funded together or separately may be decided on practical grounds [51].”

In many pilot studies, however, such criteria may be best viewed as guidelines rather than strict thresholds that determine progression. In the example, the authors found that only 24% of the family medicine groups (FMGs) agreed to participate. They state “Not reaching the pre-established criteria does not necessarily indicate unfeasibility of the trial but rather underlines changes to be made to the protocol” [34]. Clearly it is important to discuss whether such changes to protocol are likely to be feasible, and this discussion might often benefit from input independent of the trial team—for example, from the trial steering committee. This would be a reason for having such a committee in place for a pilot trial. Bugge et al. recently provided further guidance on decision making after a pilot trial [52].

In addition to the possibility of making changes to the trial protocol, investigators should also be aware that estimates of rates in pilot trials may be subject to considerable uncertainty, so that it is best to be cautious about setting definitive thresholds that could be missed simply due to chance variation [41]. In fact it is becoming increasingly common for investigators to use a traffic light system for criteria used to judge feasibility, whereby measures (eg, recruitment rates) below a lower threshold indicate that the trial is not feasible, above a higher threshold that it is feasible, and between the two that it might be feasible if appropriate changes can be made.

- Item 7a

- *Standard CONSORT item*: how sample size was determined

- *Extension for pilot trials*: rationale for numbers in the pilot trial

- *Example 1 (rationale based on assessment of practicalities and estimating rates)*

“Since this was a pilot study, a sample size calculation was not performed. The researchers aimed for 120 participants because it was felt this would be a large enough sample to inform them about the practicalities of delivering several self- management courses led by patients with COPD, recruitment, uptake, and attrition [53].”

- *Example 2 (rationale based on percentage of number required for future definitive RCT)*

“As this is a feasibility study a formal sample size calculation is not required, but we estimated the number of participants required as around 10% of the number required for the Phase 3 trial. The sample size calculation for the Phase 3 trial suggests we need to recruit 1665 participants. Given the participant population, a high level of attrition may be anticipated. We therefore aim to recruit 200 participants to the feasibility trial to inform the design and sample size of the Phase 3 RCT [54].”

- *Explanation*

The criterion of congruency between the objectives and the sample size holds as true for a pilot trial as for any study. Many pilot trials have key objectives related to estimating rates of acceptance, recruitment, retention, or uptake (see item 2b for examples). For these sorts of objectives, numbers required in the study should ideally be set to ensure a desired degree of precision around the estimated rate, although in practice it may be difficult to achieve these numbers. Additionally, for pilot trials where the key objective focuses on the acceptability or feasibility of introducing the intervention, it might be useful to consider how many sites are needed, as the acceptability or feasibility of introduction can sometimes depend on the site. In example 1, the authors state their reason for choosing their required sample size in relation to estimating rates and to exploring practicalities of implementing the intervention. They could, however, have provided stronger justification for their chosen number, such as likely recruitment or attrition rate and desired precision around these rates, so that the reader (and funder) has more grounds for believing the trial could achieve its objectives beyond a feeling.

Most methodological papers that focus on recommendations about sample size requirements for pilot trials assume that the main aim of such a trial is to estimate a quantitative measure such as the variance (or standard deviation) of an effect size to inform the sample size calculation for a future definitive RCT. Methods focus on the precision with which such estimates can be obtained. There are several relevant papers [55–57]. Among these, Whitehead et al. suggests that the size of a pilot trial should be related to the size of the future definitive RCT [58]. For such a trial designed with 90% power and two sided 5% significance, they recommend pilot trial sample sizes for each treatment arm of 75, 25, 15, and 10 for standardised effect sizes that are extra small (0.1), small (0.2), medium (0.5), or large (0.8), respectively.

Example 2 illustrates another approach that uses a sample that is a certain percentage of the expected size of the future definitive RCT. The authors reference the paper by Cocks and Torgerson, which is based on using a sample size under which a one sided 80% confidence interval for the effect size will exclude the minimum clinically important difference if the null hypothesis is true [59]. This is a similar calculation to that used in estimating sample size needed for efficacy or effectiveness but allows for additional uncertainty in the resulting effect size estimate, thus effectively assessing potential effectiveness. If an objective is to assess potential effectiveness using a surrogate or interim outcome, investigators will need to use a standard sample size calculation to ensure there is adequate power. However, this type of objective is rare in pilot trials.

- Item 7b

- *Standard CONSORT item*: when applicable, explanation of any interim analyses and stopping guidelines

- *Example*

“The board members were instructed to perform an interim analysis after 60 patients had been enrolled, at which point they could recommend stopping the trial if an overwhelming effect was detected on the basis of the critical significance level (*P* ≤ 0.02), as adjusted for the Lan– DeMets alpha-spending function with Pocock boundary” [20]

- *Explanation*

As pilot trials are small, it is uncommon for them to define criteria for early stopping, but if they do, these should be reported. The example is a pilot trial testing a surrogate outcome. There was considerable uncertainty about the variability of this outcome measure, and so the authors calculated a conservative sample size but included an interim analysis after recruiting 60 patients, in case their a priori estimates were too large and they had enough information at that stage to inform subsequent trials.

- Item 8a

- *Standard CONSORT item*: method used to generate the random allocation sequence

- *Example*

“Participants were randomly allocated to the intervention ‘MBCT group’ or ‘wait-list control group’…Random allocation was computer generated [46].”

- *Explanation*

Randomisation induces unpredictability in the allocation of each unit of randomisation. This is an important element of ensuring an unbiased treatment effect in RCTs evaluating effectiveness or efficacy because in the long run it ensures balance in characteristics between intervention groups. In a pilot trial, the soundness of the randomisation method might not directly influence robustness of the pilot trial results, which are not focused on estimates of effectiveness or efficacy, but a clear description of the process of randomisation is still important for transparent reporting.

In addition, in some pilot trials one of the objectives might be to assess the feasibility of randomisation; it is also important, therefore, that details are reported. If assessing feasibility involves more than one method being used to generate a random allocation sequence, each method should be described adequately.

- Item 8b

- *Standard CONSORT item*: type of randomisation; details of any restriction (such as blocking and block size)

- *Extension for pilot trials*: type of randomisation(s); details of any restriction (such as blocking and block size)

- *Example 1 (example with blocking)*

“Participants were randomised in block sizes of three by computer-generated randomisation to the hydration group or the control group (2:1), stratified by gender [42].”

- *Example 2 (two different types of randomisation)*

“In addition to random allocation to one of the three treatment arms, we used a 2 × 2 factorial design to distribute practices and participants across two trial design factors: cluster versus individual allocation and systematic versus opportunistic recruitment (see Fig. 1). We randomly assigned 24 practices (8 practices in each of 3 geographical regions (Bristol, Devon and Coventry)) in a 3:1 ratio to cluster (practice) allocation or individual allocation, and in a 1:1 ratio to opportunistic or systematic recruitment. The differential allocation ratio with regard to randomisation method was due to the need to ensure even numbers of practices and participants in each of the three arms across the cluster randomised practices [60].”

- *Explanation*

The type of randomisation, including whether simple or restricted, should be reported.

For practical reasons simple randomisation is sometimes used in pilot trials even when restricted randomisation is expected to be used in the future definitive RCT, and if this is the case this needs to be described.

Restricted randomisation is particularly useful in small trials evaluating the effectiveness of an intervention, to ensure balance in certain characteristics between intervention and control groups (see main CONSORT statement) [2, 61]. In pilot trials, restricted randomisation might be used to mimic the type of randomisation expected in the future definitive RCT or, if it is deemed important, to have balanced groups even if restricted randomisation is not expected to be used in the future definitive RCT. In example 1, stratified randomisation, employing blocking, was used.

One of the objectives of a pilot trial might be to assess the feasibility of randomisation; it is therefore possible that different types of randomisation could be tried, as in example 2 where cluster versus individual randomisation was considered [60].

- Item 9

- *Standard CONSORT item*: mechanism used to implement the random allocation sequence (such as sequentially numbered containers), describing any steps taken to conceal the sequence until interventions were assigned

- *Example*

“Allocation…was implemented using an automated telephone randomization service provided by the Bristol Randomized Trials Collaboration to ensure concealment from clinical staff undertaking recruitment [62].”

- *Explanation*

Ensuring allocation concealment is a cornerstone of a good randomised trial design. This mechanism performs a key function in minimising bias by preventing foreknowledge of treatment assignment, which could influence those who enrol participants. In a future definitive RCT a single mechanism will be used to conceal allocation. However, in a pilot trial the main purpose of using an allocation concealment mechanism is to establish the feasibility of the mechanism. If there is considerable uncertainty about the mechanism to be used, more than one mechanism may be tried in the pilot trial. We would expect this to be rare, but when it does occur the details of each mechanism tried should be fully described.

- Item 10

- *Standard CONSORT item*: who generated the random allocation sequence, who enrolled participants, and who assigned participants to interventions

- *Example 1 (who generated the random allocation sequence)*

“An independent statistical consultant set up the web-based randomization process to assign eligible participants to intervention or control groups by remote allocation, using permuted blocks of sizes 2 and 4. No one directly involved in the project had access to allocation codes [21].”

*Example 2 (who enrolled participants, who assigned participants to interventions)*

“Eligible children and their families were identified by the clinician conducting the assessment. If the child and his or her family were willing to find out more about the study a researcher contacted the family and arranged to visit them at a convenient location (usually at home)…Those willing to take part were randomized to receive either specialist medical care or to specialist medical care plus the Phil Parker Lightning Process (LP). Allocation…was implemented…by the Bristol Randomized Trials Collaboration…” [62]

- *Explanation*

It is important that the pilot trial confirms that allocation concealment can be implemented in a way that could be replicated in the future definitive RCT. This involves knowing who generated the randomisation sequence and who enrolled and assigned participants.

- Item 11a

- *Standard CONSORT item*: if done, who was blinded after assignment to interventions (eg, participants, care providers, those assessing outcomes) and how

- *Example 1 (blinding of multiple people)*

“Patients, families, ICU [intensive care unit] staff, ultrasound technologists, and research personnel were all blinded to drug allocation. The study pharmacist at each center was the only person who was not blinded [63].”

- *Example 2 (placebo controlled)*

“A synbiotic formulation (Synbiotic 2000®) containing 4 strains of probiotic bacteria (10^10^ each) plus 4 nondigestible, fermentable dietary fibers (2.5 g each) was provided each day, versus a fiber-only placebo formulation [64].”

- *Explanation*

In the future definitive RCT investigators will want to reduce the chance of a biased result as much as possible. Blinding is seen as one of the most effective ways of doing this, at least in trials where blinding is feasible (see main CONSORT statement for details). The main purpose of a pilot trial is to assess the feasibility of methods, including those to reduce bias. In some pilot trials it might be useful to report the method of blinding in detail, as in example 2, to help readers who might want to replicate the method in future RCTs.

It is tempting in a pilot trial to try and assess the success of blinding by asking people whether they believed they were blinded or not. This was done, for example in Arnold et al. [65]. This is not recommended, however, because evidence suggests that results of doing this largely reflects the effectiveness of the intervention rather than anything else [66].

- Item 11b

- *Standard CONSORT item*: if relevant, description of the similarity of interventions

- *Example*

“Each study drug infusion was administered using a standard volume-based rate escalation protocol preceded by the administration of 100 mg of hydrocortisone intravenously, 50 mg of diphenhydramine orally or intravenously, and 650 mg of acetaminophen orally to minimize infusion-related reactions and avoid unblinding [65].”

- *Explanation*

If blinding is done by creating a placebo, it is important in trials assessing the effect of an intervention to detail what features of the placebo were made similar to the active intervention (usually a drug)—for example, appearance, taste, smell, method of being administered. However, many of the interventions described in pilot trials are not drug interventions. Nevertheless, it remains important to describe what was done to try and ensure that the intervention and control arms received identical treatment aside from the active ingredient where this is possible. It is equally important to note that for complex interventions it might not be possible or feasible to blind certain people to allocation using these types of methods.

- Item 12a

- *Standard CONSORT item*: statistical methods used to compare groups for primary and secondary outcomes

- *Extension for pilot trials*: methods used to address each pilot trial objective whether qualitative or quantitative

- *Example 1 (descriptive and narrative reporting)*

“The feasibility outcomes were reported descriptively and narratively. For the clinical endpoints, only descriptive statistics, mean (standard deviation) for continuous outcomes and raw count (%) for categorical outcomes, were reported [67].”

- *Example 2 (confidence intervals)*

“For the primary outcomes, the feasibility criteria were the recruitment rate and duration, retention rate, safety, adverse events, compliance, acceptability of the interventions and fatigue…The recruitment rate, consisting of the eligibility and consent rate, was calculated with 95% CI…Medians (range) were reported for ordinal data (fatigue), mean (95% confidence interval (CI)) were reported for continuous data (walking speed and walking distance) and raw count (number, %) was reported for nominal data. Due to the nature of this feasibility study, it was decided not to conduct any efficacy statistical tests on the walking and fatigue data [68].”

- *Explanation*

A range of methods can be used to address the objectives in a pilot trial. These need not be statistical. Providing information about the methods used ensures that findings can be verified on the basis of the description of the analyses used. The primary focus is on methods for dealing with feasibility objectives. These methods are often based on descriptive statistics such as means and percentages but might also be narrative descriptions (example 1). Typically, any estimates of effect using participant outcomes as they are likely to be measured in the future definitive RCT would be reported as estimates with 95% confidence intervals without *P* values—because pilot trials are not powered for testing hypotheses about effectiveness.

- Item 12b

- *Standard CONSORT item*: methods for additional analyses, such as subgroup analyses and adjusted analyses

- *Extension for pilot trials*: not applicable

- *Explanation*

In a definitive trial, analyses of a difference in treatment effect for subgroups or analysis of outcomes adjusted for baseline imbalance might provide useful information. However, such analyses in a pilot trial are not applicable because the primary focus is not on determining treatment effects or differences in effects between subgroups. Rather, the focus is on assessing feasibility or piloting procedures to inform the design of the future definitive RCT.

### Results

- Item 13a

- *Standard CONSORT item*: for each group, the numbers of participants who were randomly assigned, received intended treatment, and were analysed for the primary outcome

- *Extension for pilot trials*: for each group, the numbers of participants who were approached and/or assessed for eligibility, randomly assigned, received intended treatment, and were assessed for each objective

- *Example*

**Fig 4:**
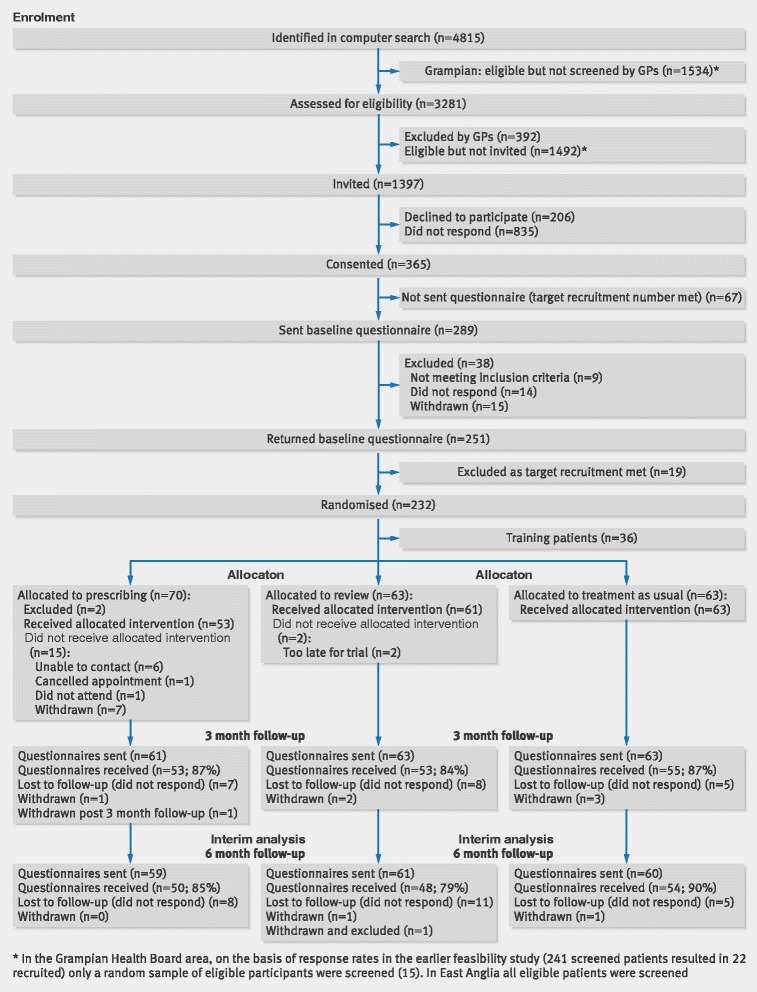
Flow diagram of a randomised pilot trial of pharmacist led management of chronic pain in primary care (reproduced from Bruhn et al [69])

**Fig 5:**
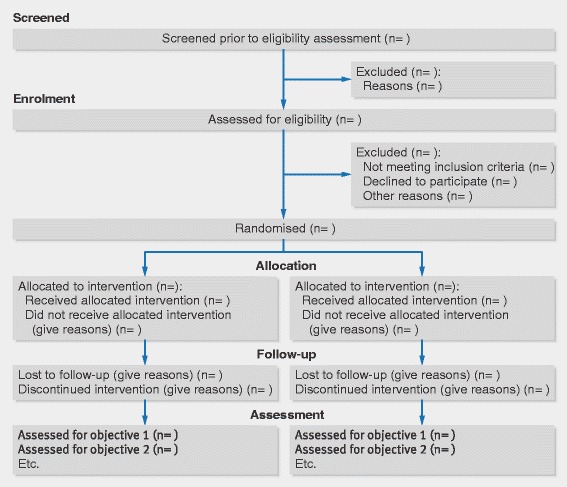
Recommended flow diagram of progress through phases of a parallel randomised pilot trial of two groups—that is, screening, enrolment, intervention allocation, follow-up, and assessed for each pilot trial objective. Adapted from Moher et al [2]

See Figs. 4 and 5 [2, 69].

- *Explanation*

As for other trials, we recommend a diagram for communicating the flow of participants in a pilot trial. A flow diagram is a key element of the CONSORT statement and has been widely adopted [70]. A review of RCTs published in five leading general and internal medicine journals found that reporting was considerably more thorough in articles that included a diagram of the flow of participants through a trial, as recommended by CONSORT [70]. A complete CONSORT flow diagram also reduces the time for readers to find essential information to assess the reliability of a trial. It is also likely to improve the availability of some information that otherwise might not be reported.

Information required to complete a CONSORT flow diagram includes the number of participants evaluated for potential enrolment into the trial and the numbers of participants who were randomly assigned to each intervention group, received treatment as allocated, completed treatment as allocated, and were analysed for the primary outcome, with numbers and reasons for exclusions at each step [2, 61].

For pilot trials it might also be important to know the number of participants who were approached (or screened) before being assessed for eligibility for potential enrolment into the trial. This ensures that readers can assess external validity and how representative the trial participants are likely to be compared with all eligible participants [71]. Additionally, for pilot trials it is important to know how many participants were approached before being evaluated for potential enrolment in the trial and how easy it was to recruit them, in order to assess the potential for enrolment for the future definitive RCT and other future trials. In some cases where these elements are a major focus of a pilot trial more information may be needed in the flow diagram (Fig. 4).

For pilot trials it is appropriate to report the number of participants assessed for each pilot trial objective, rather than the number analysed for the primary outcome (as would be the case for the future definitive RCT). If there are a limited number of objectives in the pilot trial then all should be listed and results for each objective reported in the flow diagram. If there are multiple objectives, then agreement should be reached a priori about which are the most important to decide whether to proceed to a future definitive RCT, and only these objectives should be reported in the flow diagram. Figure 5 provides a template for a CONSORT flow diagram for pilot trials, including presentation of results for different objectives. The exact form and content might, however, vary in relation to the specific features of the trial. Authors should ensure that their flow diagram matches the key objectives as far as possible.

- Item 13b

- *Standard CONSORT item*: for each group, losses and exclusions after randomisation, together with reasons

- *Example*

“All 16 patients randomised to the Symptoms Clinic attended the first appointment and 11 completed either three or four appointments. Of the remainder, two were clearly improving at the time they were seen and agreed to early discharge; two found further attendance difficult after a second appointment and one declined any further contact after the first appointment. Several patients randomised to usual care expressed some disappointment at the time of their allocation, although follow-up response rates were comparable between the two groups [72].”

- *Explanation*

For some RCTs the flow of participants through each phase of the trial can be relatively straightforward to describe, particularly if there were no losses to follow-up or exclusions. However, in more complex trials, it might be difficult for readers to identify whether and why some participants did not receive the treatment as allocated, were lost to follow-up, or were excluded [73]. In a definitive trial this information is crucial for interpreting generalisability, as participants who are excluded after allocation are unlikely to be representative of all participants in the study [74]. In a pilot trial, this information could be used to judge potential generalisability of the future definitive RCT but also to assess the acceptability of an intervention to participants and to aid planning of the future definitive RCT and other trials in similar settings.

- Item 14a

- *Standard CONSORT item*: dates defining the periods of recruitment and follow-up

- *Example*

“Patient enrolment started in August 2003 and was completed in October 2005 [75].”

- *Explanation*

It is important to report dates for all studies for transparency. An added rationale for pilot trials is that factors such as disease definitions, treatment options, and reimbursement plans that could affect the future definitive RCT might have changed between the date that the pilot trial was conducted and the date the future definitive RCT starts. The availability of different treatments outside the trial can also change and might make a difference to people’s willingness to be randomised. Thus recruitment to a pilot trial could be easier, or more difficult, than recruitment to the future definitive RCT. In addition, knowing the length of time over which the study took place might be important for planning the future definitive, and other, RCTs.

- Item 14b

- *Standard CONSORT item*: why the trial ended or was stopped

- *Extension for pilot trials*: why the pilot trial ended or was stopped

- *Example 1 (stopped without reaching intended recruitment but provided sufficient data)*

“Enterotoxigenic Escherichia coli (ETEC) is a major cause of travellers’ diarrhoea…We designed this phase II, double-blind, randomised placebo-controlled study to investigate the epidemiology of natural infection with ETEC in placebo recipients with a planned enrolment of 300 individuals, at a placebo-to-LT patch ratio of 2:1 … The study was halted when enrolment reached 201, because the planned interval for conduct had been exceeded, and it was thought that a placebo group greater than 100, although less powerful than the original 200, would be sufficient to assess the ETEC attack rate in placebo recipients…24 (22%) of 111 placebo recipients had diarrhoea, of whom 11 (10%) had ETEC diarrhoea [76].”

- *Example 2 (stopped at end of recruitment but did not provide sufficient data)*

“Recruitment rates were lower than expected which led to the study being expanded to further areas and opened to self-referral via advertisement. However, because of better management of hypertension due to changes in the UK Quality and Outcomes Framework guidelines for blood pressure treatment, few eligible patients were identified and the study closed at the end of the recruitment period, with 13 participants consenting, but 12 failing screening resulting in one recruited participant [77].”

- *Explanation*

When pilot trials end or are stopped, it is important to state why as this might affect the feasibility of the future definitive RCT. In example 1 the investigators had run out of time and thought they would have sufficient participants to estimate the rate of diarrhoea so as to inform future studies. It is not uncommon for changes in the clinical environment to occur, leading to fewer patients with unmanaged disease, and this can lead to major studies, not just pilot studies, failing to recruit. This illustrates a benefit of a pilot study to assess the likely accrual for a future definitive RCT. In example 2 the reason for stopping was simply a failure to recruit, and the reasons for this are clearly stated. Other potential reasons for stopping include the intervention being impossible to implement, other studies indicating that the research has become irrelevant, and difficulties with funding. It is also helpful to know who made the decision to stop early. In definitive RCTs a data monitoring committee often makes recommendations to stop the trial. It might not be necessary to have data monitoring committees for all pilot trials, but investigators should give some thought as to how the decision to stop should be made.

- Item 15

- *Standard CONSORT item*: a table showing baseline demographic and clinical characteristics for each group

- *Example*

See Table 4.

- *Explanation*

In an RCT evaluating the effect of an intervention, a table of baseline characteristics is important to indicate any differences between intervention groups that could affect the face validity of the trial. In a pilot trial, the number of participants is likely to be smaller than in the future definitive RCT and baseline imbalances might therefore be more likely. Similar to a definitive trial, imbalance does not suggest bias, and in any case bias is not a problem in the same way it is in a definitive trial because an assessment of the effect of an intervention is not the primary concern. Nevertheless, baseline data are important to aid interpretation of the results, including a consideration of generalisability, and a table is the best way of presenting this information.

- Item 16

*Standard CONSORT item*: for each group, number of participants (denominator) included in each analysis and whether the analysis was by original assigned groups

- *Extension for pilot trials*: for each objective, number of participants (denominator) included in each analysis. If relevant, these numbers should be by randomised group

- *Example 1 (number of sites contacted)*

“A research assistant made 41 introductory phone calls to contact the medical directors of the 21 eligible FMGs [family medicine groups] over a four-week period. One director could not be contacted. Information leaflets were faxed to the 20 contacted FMGs [34].”

- *Example 2 (number of practitioners taking part within sites)*

“Out of the 52 eligible family physicians working in the five participating FMGs [family medicine groups], 39 (75%) agreed to participate in the study [34].”

- *Explanation*

In RCTs evaluating the effect of an intervention, outcomes are usually measured on participants and therefore denominators are numbers of participants. However, because of the potential variety of objectives in a pilot trial, the denominators for measures that assess feasibility according to these objectives might be organisations, health practitioners, patients, or, in some cases, episodes or events. In the interests of simplicity we have not changed the word “participants” in this item, but the item should be interpreted in the light of the particular objective and associated measure or assessment. The two examples are taken from the same trial. One objective was to assess the feasibility of recruitment. Participants for that objective are FMGs (example 1) and family physicians (example 2). The denominators of 21 (FMGs) and 52 (family physicians) indicate numbers approached and therefore the effort involved in recruiting. In this example providing numbers by randomised group is not relevant.

- Item 17a

- *Standard CONSORT item*: for each primary and secondary outcome, results for each group, and the estimated effect size and its precision (such as 95% confidence interval)

- *Extension for pilot trials*: for each objective, results including expressions of uncertainty (such as 95% confidence interval) for any estimates. If relevant, these results should be by randomised group

- *Example 1 (feasibility outcome)*

“The ABSORB [A bioabsorbable everolimus-eluting coronary stent system for patients with single de-novo coronary artery lesions] study aimed to assess the feasibility and safety of the BVS [bioasorbable everolimus-eluting stent] stent in patients with single de-novo coronary artery lesions…Procedural success was 100% (30/30 patients), and device success 94% (29/31 attempts at implantation of the stent) [78].”

- *Example 2 (proposed outcome in future definitive trial)*

“Rates of initiation of lifestyle change also favoured the individualized assessment arm but less clearly. At 3 months, 75% of the individualized assessment arm and 68% of the usual assessment arm had initiated changes in their lifestyle (unadjusted odds ratio, 1.38 [95%CI, 0.55 to 3.52]). At 6 months, the percentages were 85 and 75%, suggesting increased initiation of change over time in both arms, with the gap widening slightly (unadjusted odds ratio, 1.86 [95% CI, 0.64 to 5.77])…Wide CIs again point to the degree of uncertainty around this conclusion” [40]

- *Explanation*

It is important that the reported results of a pilot trial reflect the objectives. Results might include, for example, recruitment, retention or response rates, or other sorts of rates, as in example 1. Because the sample size in a pilot trial is likely to be small, estimates of these rates will be imprecise and this imprecision should be recognised, for example, by calculating a confidence interval around the estimate. Commonly, authors do not give such a confidence interval, but if the numerator and denominator are given the confidence interval can be calculated. In example 1 the Wilson 95% confidence interval for 100% (30/30) is 88.65 to 100% and for 94% (29/31) is 79.78 to 98.21% (OpenEpi Seattle) [78]. If authors do report differences between trial arms (and this is not necessary if it is not consistent with the objectives of the trial) then confidence intervals again provide readers with an assessment of precision (example 2), which usually indicates considerable uncertainty. If samples in the pilot trial and future definitive RCT are drawn from slightly different populations, confidence intervals calculated from the pilot will not directly indicate the likely upper and lower bounds of the relevant measure in the future definitive RCT, but can nevertheless highlight the lack of precision effectively.

- Item 17b

- *Standard CONSORT item*: for binary outcomes, presentation of both absolute and relative effect sizes is recommended

- *Extension for pilot trials*: not applicable

- *Explanation*

This item is included in the 2010 CONSORT statement because when considering clinical implications, neither the relative nor the absolute measures of effect size for binary outcomes give a complete picture of the effect of an intervention. For example, relative risks are less affected by differences in baseline populations across studies than are absolute risks, although sometimes can be misinterpreted in terms of population benefit. In addition, different audiences (clinical, policy, patient) prefer to use one or the other measure. However, in pilot trials the situation is different. Because of the imprecision of estimates from these trials and the fact that samples in these trials can be unrepresentative (see item 17a), we caution against any reliance on estimates of effect size from pilot trials for clinical implications (see also Introduction, Scope of this paper, and Box 1). Information from outcome data, however, can be legitimately used for other purposes, such as estimating inputs for sample size for the future definitive RCT (see item 7a). Thus item 17b, which is underpinned by rationale around clinical implications, is not applicable.

- Item 18

- *Standard CONSORT item*: results of any other analyses performed, including subgroup analyses and adjusted analyses, distinguishing prespecified from exploratory

- *Extension for pilot trials:* results of any other analyses performed that could be used to inform the future definitive RCT

- *Example*

“Sensitivity analysis

At both six and 12 weeks, findings were insensitive to the exclusion of those catheterised throughout their hospital stay (and also to the exclusion of those who were never incontinent following the removal of a catheter). However, at both time points, odds ratios reduced when those with pre-stroke incontinence were excluded…” [79]

- *Explanation*

It is possible that the results of analyses that were not initially planned might have important implications for the future definitive RCT. Such findings should be reported and discussed in relation to how they might inform the future definitive RCT. In the example, although numbers were small, the authors inferred from the unplanned sensitivity analyses that those with pre-stroke incontinence were at least as likely, or more likely, to benefit from the intervention than those continent pre-stroke, and concluded that this group of patients should be included in the full trial.

- Item 19

- *Standard CONSORT item*: all important harms or unintended effects in each group (for specific guidance see CONSORT for harms) [7]

- *Example 1 (potential harm)*

“Intervention and usual treatment groups were similar in terms of age, gender, and marital status, but those in the intervention group were more likely to be unemployed (69 v. 59%), to use methods other than poisoning (23 v. 9%), to have a past history of self-harm (67 v. 53%) and to have had previous psychiatric treatment (64 v. 53%).

Online Table DS1 shows self-harm repetition and resource use in the two groups. The 12-month repeat rate for individuals in the intervention group was 34.4 v. 12.5% for the usual treatment group (odds ratio (OR) 3.67, 95% CI 1.0–13.1 …)…Adjusting for baseline clinical factors (centre, method of harm (self-poisoning v. other), previous self-harm, previous psychiatric treatment), the odds ratio for repetition and incidence rate ratio for number of repeat episodes remained elevated…” [80]

- *Example 2 (unintended effect or potential harm)*

“An unanticipated finding in this study was a 4-kg weight loss, on average, favouring the intervention group, although we recognized that there were some differences in weight between groups at study commencement that may have had an effect on our results…Thus, there is a clear role for dietary considerations in any study that aims to positively influence body weight. Although we provided one educational session on nutrition during a tour of a local grocery store with a dietitian and modelled healthy food choices with the lunches provided, dietary behaviors and body weight were not the focus of the study [21].”

- *Explanation*

It is crucial to report all important or potential harms or unintended effects on individual participants in each group to enable the study design for the future definitive RCT to be changed either to avoid these effects or to put in place effective processes for monitoring potential harms. In example 1, it was not clear whether the unexpected increased risk of repeated self harm in the intervention group was real or a consequence of baseline covariate imbalance, or peculiar to the particular setting. This led to a proposal to change the design to use stratified randomisation in the future definitive RCT. In example 2, the unintended effect of weight loss in elderly participants led to the decision to include a dietary component in the intervention to avoid potential harm in the future definitive RCT. This information might also be useful to other researchers planning similar studies.

- Item 19a

- *Extension for pilot trials*: if relevant, other important unintended consequences

- *Example (unintended consequence)*

“Twelve of the 13 active, and 11 of the 13 traditional practices recruited a total of 231 participants in the 12 months from mid-April 1998. Active practices recruited 165 (average practice recruitment rate of 1.71 per 1000 registered patients, i.e., 141% of expected) while traditional practices recruited only 66 (0.57 per 1000 i.e. 54% of expected) (Fig. 1). On average active practices recruited 12.7 participants (range 0–39), while traditional practices recruited only 5.1 participants (range 0–18) (Table 2). Although both types of practices recruited similar percentages of those identified (13% in active; 16% in traditional), active identified 1257, far more than the 416 by traditional practices. The extreme difference in recruitment rates led to an investigation of baseline characteristics of participants in the two groups (Table 3). Participants recruited by active practices were more likely to be working full-time and to have had further education since leaving school. They were also suffering from milder back pain, less limited physically and less depressed [81].”

**Table 4:** Example of baseline information for each group. From Seebacher et al [68]

Parameter	Group A	Group B	Group C
	Music cued motor imagery	Metronome cued motor imagery	Control group
	(*n* = 10)	(*n* = 10)	(*n* = 10)
Females to males	10:0	7:3	5:5
Age (years)^a^	47.3 (38.4, 56.2)	41.8 (34.8, 48.8)	46.1 (39.8, 52.5)
EDSS^b^	3 (1.5, 4.5)	2.5 (1.5, 4.5)	2.5 (1.5, 4.0)
MFIS total score^b^	35 (3, 67)	32 (17, 50)	33.5 (0, 48)
Participants with fatigue (MFIS total score ≥38)	4/10	2/10	4/10
T25FW (s)^a^	6.1 (4.5, 7.6)	5.4 (4.5, 6.2)	5.2 (4.3, 6.1)
6MWT (m)^a^	453.1 (365.0, 541.1)	428.2 (352.8, 503.6)	484.7 (399.5, 569.8)

*EDSS* Expanded Disability Status Scale, *MFIS* Modified Fatigue Impact Scale, *T25FW* Timed 25-Foot Walk, *s* seconds, *6MWT* 6-Minute Walk Test, *m* metres

^a^Mean (95% confidence interval)

^b^Median (range)

- *Explanation*

This is a new item reflecting the importance of reporting unintended consequences that do not directly affect individual participants but might have implications for the validity of the future definitive RCT if not dealt with in the pilot trial. By unintended consequences we mean things that happened in the pilot trial that the investigators did not intend to look for but that would have such implications. In the example, the design of the pilot trial included practice level randomisation, with participant recruitment after that randomisation. This had unintended consequences in the balance of recruited participants between arms, and in the main study the researchers abandoned randomisation at the practice level.

### Discussion

- Item 20

- *Standard CONSORT item*: trial limitations, addressing sources of potential bias, imprecision, and, if relevant, multiplicity of analyses

- *Extension for pilot trials*: pilot trial limitations, addressing sources of potential bias and remaining uncertainty about feasibility

- *Example 1 (pilot trial limitations)*

“In some cases, platelet mass was calculated on an MPV [mean platelet volume] that was up to 72 hours old based on our previous research on the relationship between platelet mass and IVH [intraventricular hemorrhage]. We cannot rule out the possibility that during acute thrombocytopenia changes in MPV may be more acute. Because platelet counts were not confirmed by manual count, we cannot exclude the unlikely possibility that some infants may have had pseudothrombocytopenia [82].”

- *Example 2 (potential bias)*

“Fourth, the house staff at the two academic centers in the study may have been a source of contamination. Additional house staff occasionally provided overnight coverage at the intervention group academic center. These additional house staff were not formally educated about the study, so they effectively functioned as if they were in the control group. Conversely, additional house staff who provided overnight coverage at the control group academic center may have been previously educated about our study while working at the intervention group academic center. Thus, they effectively functioned as if they were in the intervention group [83].”

- *Example 3 (remaining uncertainty)*

“The integration of a nested, internal pilot in the definitive trial should also be considered to allow continued monitoring of the feasibility, in particular, the assessment of using different inclusion criteria and the recommended changes to the data collection methods, particularly within the first year of recruitment. The use of a qualitative element to assess the participants’ views on data collection methods would also be beneficial [84].”

- *Explanation*

Identifying and discussing the limitations of a study helps to provide a better context for understanding the importance of its findings. In a pilot trial it might also be helpful to distinguish between limitations that can be overcome in a future definitive RCT, and those that cannot. In example 1 the authors explain the limitation of a method of measurement although they do not say whether they think this could be overcome in a future definitive RCT.

In a future definitive RCT, investigators will want, as far as possible, to avoid sources of bias that might affect treatment effect estimates. In a pilot trial, investigators are not primarily interested in treatment effect, so these biases will not be of so much concern but it would still be useful to identify potential biases that could affect the treatment effect in the future definitive RCT so that investigators have a better chance of avoiding these. In example 2 a potential source of bias in the future definitive RCT is identified.

If substantial areas of uncertainty about feasibility remain at the end of the pilot trial that prevent investigators from proceeding with a future definitive RCT or warrant investigation in an internal pilot then, for clarity, these should be reported, as in example 3.

Lastly, although we do not recommend this, if underpowered tests are performed and reported then investigators should always point out this limitation to avoid misinterpretation of results (see item 2b).

- Item 21

- *Standard CONSORT item*: generalisability (external validity, applicability) of the trial findings

- *Extension for pilot trials*: generalisability (applicability) of pilot trial methods and findings to future definitive trial and other studies

- *Example 1 (generalisability of findings)*

“We accommodated variability in choice and duration of standard treatments to enhance generalizability of the results and had high rates of follow-up.” [65]

- *Example 2 (generalisability to other pilot trials)*

“Our data reflect the activities of only one pilot trial; however, we hope that the methods may serve as a template for analyzing other pilot studies with different designs in other settings [63].”

- *Example 3 (generalisability concerns)*

“Although safety issues must remain paramount in practice and clinical research, common overstringent exclusion criteria may increase perceived trial safety yet limit the generalizability of trial results and delay answers to important clinical questions. Reevaluation of the PROTECT Pilot exclusion criteria will…enhance the applicability of the larger PROTECT study…The PROTECT Pilot indicated the need for another pilot study (DIRECT) to determine the safety of dalteparin 5000 IU SC OD among patients with severe renal insufficiency (creatinine clearance, b30 mL/min) [63].”

- *Explanation*

Generalisability (applicability) is the extent to which aspects of a study can be applied to other circumstances. Generalisability is not absolute and is a matter of judgment. In a definitive trial, readers are usually interested in the generalisability of findings to situations outside research settings—for example, routine clinical practice. However, in pilot trials this is not the case because the size of these studies does not allow this. Nevertheless, it might be important to consider generalisability at the pilot stage as this could be important for the generalisability of the future definitive RCT (example 1), the findings and the methods might be applied in research settings other than the future definitive RCT (example 2), or there might be concerns about the generalisability of results from a future definitive RCT conducted in an identical way to the pilot trial that might lead to changes in the design of the future definitive RCT or further piloting (example 3).

- Item 22

- *Standard CONSORT item*: interpretation consistent with results, balancing benefits and harms, and considering other relevant evidence

- *Extension for pilot trials*: interpretation consistent with pilot trial objectives and findings, balancing potential benefits and harms, and considering other relevant evidence

- *Example 1 (consistency with objectives and findings)*

“One of the goals of this pilot study was to investigate the feasibility of using platelet transfusion guidelines based on platelet mass. In five infants, MPV [mean platelet volume] was not available within 72 hours preceding the diagnosis of thrombocytopenia. A lack of immediately available MPV may limit the clinical utility and generalizability of this transfusion strategy at some institutions…In our study approximately half of the families at the Christiana Hospital site did not consent to the study. This information is important for planning future studies on platelet transfusion. Many families were unable to decide on enrollment at a time when their infant was thrombocytopenic and facing transfusion. An alternative study design for platelet transfusion study may involve enrolling a larger number of infants on admission, regardless of platelet count, with transfusion guidelines to apply only if they actually become thrombocytopenic. This approach may limit the stress on families of being approached about the need for transfusion and a transfusion related study simultaneously [82].”

- *Example 2 (considering other relevant evidence)*

“As far as we know, our participants were able to perform motor imagery. Our results seem to be in contrast to previous studies demonstrating a lower capacity for motor imagery in people with MS. However, these authors linked impaired motor imagery in this population particularly to cognitive dysfunction and depression. Therefore, persons with cognitive impairment and depression were excluded from our study. Several studies used patient-rated questionnaires, such as the Kinaesthetic and Visual Imagery Questionnaire to assess the motor imagery ability in their participants. Our study could have used this patient-rated questionnaire, but our participants were called weekly to ask for any problems with kinaesthetic motor imagery, and they were supported accordingly. In addition, all motor imagery ability studies in people with MS were experimental studies with no long-term training effects, in contrast to our 4 weeks duration study with 24 training sessions which might have enhanced the mental representation [68].”

- *Example 3 (consistency with findings in relation to decision criteria)*

“Moreover, the results of this trial support the feasibility and acceptability of conducting a large clustered randomised trial involving dyads of family physicians and their patients in SDM regarding the optimal use of antibiotics for ARI. This conclusion is reached even if not all predetermined standards for our criteria were always fully met. Indeed, it has been established that not reaching the preestablished criteria does not necessarily indicate unfeasibility of the trial but rather underlines changes to be made to the protocol…24% of the eligible FMGs agreed to take part in the study, less than the 50% expected. We were probably too confident when targeting a 50% positive response rate from all identified FMGs [34].”

- *Explanation*

Interpretation of findings helps increase understanding of the importance of the results. In example 1, in addition to matching their interpretation to one of the goals of the study, the authors draw out the issue of redesign to reduce stress in families approached and so increase recruitment—and hopefully eventually a positive benefit for the children involved. This observation could be helpful to others planning similar studies. As for definitive trials, readers will want to know how the evidence presented in the report of a pilot trial relates to evidence from other sources (example 2). These sources might be other feasibility studies carried out by the authors or studies by different authors in the same or similar settings or with similar patients. If a priori decision criteria have been used (item 6c) then interpretation should be made with reference to these criteria (example 3).

- Item 22a

*Extension for pilot trials*: implications for progression from pilot to future definitive trial, including any proposed amendments

- *Example 1 (proposed amendments to improve recruitment)*

“The target of recruit to time was met but this did not translate to the expected number of eligible patients being recruited. Eligibility of the screened population was much lower than expected, indicating that the inclusion criteria may have been too stringent. The exclusion criteria of BMI ≤22 kg/m2 was based on published evidence that a BMI at the lower end of the normal range can increase mortality in the haemodialysis population…However, body composition is thought to play a much greater role in the protective effects of a greater BMI, than the BMI itself…The use of BMI as a screening tool was a quick and easy measure but the level of ≤22 kg/m2 should be reassessed prior to a definitive trial. If the BMI was raised to ≤24 kg/m2 then this would have increased potential recruitment by 10% [84].”

- *Example 2 (proposed amendments to improve cooperation)*

“Six homes declined to actively participate before even beginning the intervention. To ensure cooperation by the entire team and avoid early withdrawal, a short presentation to the Professional Advisory Committee team could potentially boost recruitment/retention. Obtaining initial consent from both the medical director and director of care may also be beneficial. Furthermore, to overcome logistical challenges, particularly for homes in the far north, providing an opportunity to view modules on a Web site or participate remotely may improve participation [85].”

- *Example 3 (implications for progression to future definitive RCT)*

“Hospitals that were allocated to receive our multicomponent intervention comprising education, standardized paper-based physician orders, and group audit and feedback did not have a higher rate of hospitalized medical patients appropriately managed for thromboprophylaxis within 24 hours of admission than did hospitals that were not allocated to this strategy (63% vs. 67%). This finding, coupled with the problems associated with ensuring preprinted orders were placed in all medical charts led us to conclude that this intervention should not be provided on a larger scale without major revision and testing. That is, it was not feasible [83].”

- *Explanation*

This is a new item. To progress from a pilot trial to a future definitive RCT, it is important to understand how the implications of the findings in the pilot carry over to the future definitive RCT. To aid clarity, a simple statement as to whether the future definitive RCT will be planned without any changes from the pilot trial, planned with changes from the pilot trial (examples 1 and 2), or not planned because of major problems with feasibility (example 3), is sufficient. If it is proposed to plan the future definitive RCT with specific changes from the pilot trial, these should be stated.

### Other information

- Item 23

- *Standard CONSORT item*: registration number and name of trial registry

- *Extension for pilot trials*: registration number for pilot trial and name of trial registry

- *Example*

“Trial registration number: Clinical Trials, protocol registration system: NCT01695070 [86].”

- *Explanation*

It is just as important for a pilot trial to be registered with a unique identifier as it is for a definitive trial. Registration ensures transparency and accountability and in the United Kingdom is now a requirement for all clinical trials before approval from UK ethics committees [87, 88] It ensures all ongoing work is in the public domain, and subsequent publication (and therefore access to findings for the greater good) confirmed. The World Health Organization states that “the registration of all interventional trials is a scientific, ethical and moral responsibility [89].” The International Committee of Medical Journal Editors requires all trials to be registered as a criterion for publication and lists suggested registries [90].

- Item 24

- *Standard CONSORT item*: where the full trial protocol can be accessed, if available

- *Extension for pilot trials*: where the pilot trial protocol can be accessed, if available

- *Example 1 (reference to published protocol)*

“The Healthy Hospital Trial is a single-center, randomized controlled, 2-arm, parallel-group, unblinded feasibility trial that was conducted on 2 cardiology wards at the Leeds Teaching Hospitals Trust. Its primary aim was to explore the feasibility of individualized lifestyle referral assessment, estimate the rate of recruitment, and explore the feasibility of collecting the data and follow-up of participants to inform the sample size of a definitive trial....The trial protocol has been published elsewhere [40].”

- *Example 2 (protocol as supporting information)*

“The protocol for this trial and supporting TREND checklist are available as supporting information; see Checklist S1 and Protocol S1 [91].”

- *Example 3 (protocol available from authors on request)*

“Participants in the control arm (but not the other two arms) received a 16-page informational booklet relevant to education, medical care, housing, employment, and community resources (protocol available from authors upon request) [92].”

- *Explanation*

Access to the full protocol for the pilot trial is important as it will prespecify all the main components of the trial. The SPIRIT (standard protocol items: recommendations for interventional trials) statement defines an evidence based set of items that would be included [93]. Accessibility of the protocol allows subsequent output to be checked for completeness, and reduces the chance of selective reporting to suggest “better” results. The examples illustrate the different ways in which protocols may be made available, such as prior publication (example 1), as an addendum to the report of the pilot trial (example 2), or on request from the authors (example 3). Options where the protocol is already in the public domain, such as prior publication, are to be preferred. Other methods that could be used to achieve this would include publication on a study website. Trial registries (see item 23) also include some core protocol items.

- Item 25

- *Standard CONSORT item*: sources of funding and other support (such as supply of drugs), role of funders

- *Example*

**Funding**: “This trial was funded through grants from Academic Health Science Centres Alternative Funding Plan Innovation Fund of Ontario and Octapharma Canada. The trial funders had no role in the design of the study, the collection, analysis or interpretation of data, the writing of the report, or the decision to submit the article for publication [94].”

- *Explanation*

Reporting the sources of all funding for a pilot trial (that is, the main research award and any other support, such as supply of equipment) allows readers to judge the potential influence of the funding body on the design, conduct, analysis, and reporting of the trial. If no specific funding was provided to support the pilot trial, this should also be stated. As reported in the main CONSORT statement, a systematic review has shown that research funded by the pharmaceutical industry is more likely to report findings in its favour, compared with reports of research funded by independent funding bodies [2, 61] Where funders have had no involvement in any aspect of trial conduct or reporting this should be explicitly stated.

- Item 26

- *Extension for pilot trials*: ethical approval/research review committee approval confirmed with reference number

- *Example*

“The Regional Ethical Review Board at the Karolinska Institute approved the study, no. 2007/1401-31/3 [95].”

- *Explanation*

This is a new item that has been added to the CONSORT checklist because of the need to emphasise that all research, including pilot trials, should only be conducted within an ethical framework and with all ethical and other approvals in place before commencement. Of particular relevance to pilot trials is the need also to be aware of any restrictions imposed by the reviewing ethical committee, because these would have implications for the design and conduct of the future definitive RCT.

## Comment

Reports of RCTs need to include key information on the methods and results so that readers can accurately interpret the contents of the report. This is as true for pilot trials as it is for any other RCT. The CONSORT 2010 statement provides the latest recommendations from the CONSORT Group on essential items to be included in the report of an RCT [2, 61] However, pilot trials differ from other randomised trials in their aims and objectives, focusing on assessing feasibility rather than effectiveness or efficacy. Therefore, although much of the information to be reported in these trials is similar to that which needs to be reported in any other randomised trial, there are some key differences in the type of information and in the appropriate interpretation of standard CONSORT reporting items.

In this article we introduce and explain these key differences in an extension to the CONSORT checklist specific to pilot trials. In the section entitled “Scope of this paper” we discuss several other types of feasibility study, and “proof of concept” trials. Other researchers have begun to look at the transfer of ideas between these different types of study (eg, Wilson et al [96]). It is our expectation that some of the principles of reporting outlined in this extension can be adapted for other types of feasibility or proof of concept studies.

Use of the CONSORT statement for the reporting of two group parallel trials is associated with improved reporting quality [97]. We believe that the routine use of this proposed extension to the CONSORT statement will result in similar improvements in reporting of pilot trials. When reporting a pilot trial, authors should address each of the 26 items on the CONSORT extension checklist using this document, referring to the main CONSORT guidelines as appropriate. Adherence to the CONSORT statement and extensions can also help researchers designing trials in the future and can guide peer reviewers and editors in their evaluation of manuscripts. Many journals recommend adherence to the CONSORT recommendations in their instructions to authors. We encourage them to direct authors to this and to other extensions of CONSORT for specific trial designs. A tool is currently being developed to support journals in doing this [98]. The most up to date versions of all CONSORT recommendations are available at www.consort-statement.org (Additional file 1).

**Web extra** Extra material supplied by authors.

## **Box 1. Methodological considerations and principles that guided the development of the CONSORT extension to pilot trials**

• The rationale of a pilot trial is to investigate areas of uncertainty about the future definitive RCT

• The primary aims and objectives of a pilot trial are therefore about feasibility, and this should guide the methodology used in the pilot trial

• Assessments or measurements to address each pilot trial objective should be the focus of data collection and analysis. This might include outcome measures likely to be used in the definitive trial but, equally, it might not

• Since the aim of a pilot trial is to assess the feasibility of proceeding to the future definitive RCT, a decision process about how to proceed needs to be built into the design of the pilot trial. This might involve formal progression criteria to decide whether to proceed, to proceed with amendments, or not to proceed

• Methods used to address each pilot trial objective can be qualitative or quantitative. A mixed methods approach could result in both types of data being reported within the same paper. Equally, a process evaluation or other qualitative study can be done alongside a pilot trial and reported separately in more detail

• The number of participants in a pilot study should be based on the feasibility objectives and some rationale should be given

• Formal hypothesis testing for effectiveness (or efficacy) is not recommended. The aim of a pilot trial is not to assess effectiveness (or efficacy) and it will usually be underpowered to do this

## Additional file

Additional file 1 CONSORT checklist of information to include when reporting a pilot trial (PDF 84 kb)
