# Supplementary material for: CONSORT 2010 statement: extension to randomised pilot and feasibility trials
Source: Pilot Feasibility Stud. 2016 Oct 21;2:64. doi: 10.1186/s40814-016-0105-8 (PMC5154046; doi:10.1186/s40814-016-0105-8)
Supplement: Additional file 1 — CONSORT checklist of information to include when reporting a pilot trial (PDF 84 kb) [file 40814_2016_105_MOESM1_ESM.pdf]

CONSORT checklist of information to include when reporting a pilot trial*

| Section/topic and item No | Standard checklist item | Extension for pilot trials | Page No where item is reported |
|---|---|---|---|
| **Title and abstract** | | | |
| 1a | Identification as a randomised trial in the title | Identification as a pilot or feasibility randomised trial in the title | |
| 1b | Structured summary of trial design, methods, results, and conclusions (for specific guidance see CONSORT for abstracts) | Structured summary of pilot trial design, methods, results, and conclusions (for specific guidance see CONSORT abstract extension for pilot trials) | |
| **Introduction** | | | |
| Background and objectives: | | | |
| 2a | Scientific background and explanation of rationale | Scientific background and explanation of rationale for future definitive trial, and reasons for randomised pilot trial | |
| 2b | Specific objectives or hypotheses | Specific objectives or research questions for pilot trial | |
| **Methods** | | | |
| Trial design: | | | |
| 3a | Description of trial design (such as parallel, factorial) including allocation ratio | Description of pilot trial design (such as parallel, factorial) including allocation ratio | |
| 3b | Important changes to methods after trial commencement (such as eligibility criteria), with reasons | Important changes to methods after pilot trial commencement (such as eligibility criteria), with reasons | |
| Participants: | | | |
| 4a | Eligibility criteria for participants | | |
| 4b | Settings and locations where the data were collected | | |
| 4c | | How participants were identified and consented | |
| Interventions: | | | |
| 5 | The interventions for each group with sufficient details to allow replication, including how and when they were actually administered | | |
| Outcomes: | | | |
| 6a | Completely defined prespecified primary and secondary outcome measures, including how and when they were assessed | Completely defined prespecified assessments or measurements to address each pilot trial objective specified in 2b, including how and when they were assessed | |
| 6b | Any changes to trial outcomes after the trial commenced, with reasons | Any changes to pilot trial assessments or measurements after the pilot trial commenced, with reasons | |
| 6c | | If applicable, prespecified criteria used to judge whether, or how, to proceed with future definitive trial | |

| | | |
|---|---|---|
| Sample size: | | |
| 7a | How sample size was determined | Rationale for numbers in the pilot trial |
| 7b | When applicable, explanation of any interim analyses and stopping guidelines | |
| Randomisation: | | |
| Sequence generation: | | |
| 8a | Method used to generate the random allocation sequence | |
| 8b | Type of randomisation; details of any restriction (such as blocking and block size) | Type of randomisation(s); details of any restriction (such as blocking and block size) |
| Allocation concealment mechanism: | | |
| 9 | Mechanism used to implement the random allocation sequence (such as sequentially numbered containers), describing any steps taken to conceal the sequence until interventions were assigned | |
| Implementation: | | |
| 10 | Who generated the random allocation sequence, enrolled participants, and assigned participants to interventions | |
| Blinding: | | |
| 11a | If done, who was blinded after assignment to interventions (eg, participants, care providers, those assessing outcomes) and how | |
| 11b | If relevant, description of the similarity of interventions | |
| Analytical methods: | | |
| 12a | Statistical methods used to compare groups for primary and secondary outcomes | Methods used to address each pilot trial objective whether qualitative or quantitative |
| 12b | Methods for additional analyses, such as subgroup analyses and adjusted analyses | Not applicable |
| **Results** | | |
| Participant flow (a diagram is strongly recommended): | | |
| 13a | For each group, the numbers of participants who were randomly assigned, received intended treatment, and were analysed for the primary outcome | For each group, the numbers of participants who were approached and/or assessed for eligibility, randomly assigned, received intended treatment, and were assessed for each objective |
| 13b | For each group, losses and exclusions after randomisation, together with reasons | |
| Recruitment: | | |

| | | |
|---|---|---|
| 14a | Dates defining the periods of recruitment and follow-up | |
| 14b | Why the trial ended or was stopped | Why the pilot trial ended or was stopped |
| Baseline data: | | |
| 15 | A table showing baseline demographic and clinical characteristics for each group | |
| Numbers analysed: | | |
| 16 | For each group, number of participants (denominator) included in each analysis and whether the analysis was by original assigned groups | For each objective, number of participants (denominator) included in each analysis. If relevant, these numbers should be by randomised group |
| Outcomes and estimation: | | |
| 17a | For each primary and secondary outcome, results for each group, and the estimated effect size and its precision (such as 95% confidence interval) | For each objective, results including expressions of uncertainty (such as 95% confidence interval) for any estimates. If relevant, these results should be by randomised group |
| 17b | For binary outcomes, presentation of both absolute and relative effect sizes is recommended | Not applicable |
| Ancillary analyses: | | |
| 18 | Results of any other analyses performed, including subgroup analyses and adjusted analyses, distinguishing prespecified from exploratory | Results of any other analyses performed that could be used to inform the future definitive trial |
| Harms: | | |
| 19 | All important harms or unintended effects in each group (for specific guidance see CONSORT for harms) | |
| 19a | | If relevant, other important unintended consequences |
| **Discussion** | | |
| Limitations: | | |
| 20 | Trial limitations, addressing sources of potential bias, imprecision, and, if relevant, multiplicity of analyses | Pilot trial limitations, addressing sources of potential bias and remaining uncertainty about feasibility |
| Generalisability: | | |
| 21 | Generalisability (external validity, applicability) of the trial findings | Generalisability (applicability) of pilot trial methods and findings to future definitive trial and other studies |
| Interpretation: | | |
| 22 | Interpretation consistent with results, balancing benefits and harms, and considering other relevant evidence | Interpretation consistent with pilot trial objectives and findings, balancing potential benefits and harms, and considering other relevant evidence |
| 22a | | Implications for progression from pilot to future definitive trial, including any proposed amendments |
| **Other information** | | |

| | | | |
|---|---|---|---|
| Registration: | | | |
| | 23 | Registration number and name of trial registry | Registration number for pilot trial and name of trial registry |
| Protocol: | | | |
| | 24 | Where the full trial protocol can be accessed, if available | Where the pilot trial protocol can be accessed, if available |
| Funding: | | | |
| | 25 | Sources of funding and other support (such as supply of drugs), role of funders | |
| | 26 | | Ethical approval or approval by research review committee, confirmed with reference number |

*Here a pilot trial means any randomised study conducted in preparation for a future definitive RCT, where the main objective of the pilot trial is to assess feasibility.
